# Standardization procedures for real-time breath analysis by secondary electrospray ionization high-resolution mass spectrometry

**DOI:** 10.1007/s00216-019-01764-8

**Published:** 2019-04-15

**Authors:** Kapil Dev Singh, Georgi Tancev, Fabienne Decrue, Jakob Usemann, Rhea Appenzeller, Pedro Barreiro, Gabriel Jaumà, Miriam Macia Santiago, Guillermo Vidal de Miguel, Urs Frey, Pablo Sinues

**Affiliations:** 1 University Children’s Hospital Basel, University of Basel, Spitalstr. 33, 4056 Basel, Switzerland; 2 Department of Biomedical Engineering, University of Basel, Gewerbestr. 14, 4123 Allschwil, Switzerland; 3 Fossil Ion Technology S.L., Calle la Gitanilla 17, 29004 Malaga, Spain

**Keywords:** Breath metabolomics, Fatty aldehydes, Secondary electrospray ionization high-resolution mass spectrometry, Oxidative stress, Standardization procedures, Variability

## Abstract

Despite the attractiveness of breath analysis as a non-invasive means to retrieve relevant metabolic information, its introduction into routine clinical practice remains a challenge. Among all the different analytical techniques available to interrogate exhaled breath, secondary electrospray ionization high-resolution mass spectrometry (SESI-HRMS) offers a number of advantages (e.g., real-time, yet wide, metabolome coverage) that makes it ideal for untargeted and targeted studies. However, so far, SESI-HRMS has relied mostly on lab-built prototypes, making it difficult to standardize breath sampling and subsequent analysis, hence preventing further developments such as multi-center clinical studies. To address this issue, we present here a number of new developments. In particular, we have characterized a new SESI interface featuring real-time readout of critical exhalation parameters such as CO_2_, exhalation flow rate, and exhaled volume. Four healthy subjects provided breath specimens over a period of 1 month to characterize the stability of the SESI-HRMS system. A first assessment of the repeatability of the system using a gas standard revealed a coefficient of variation (CV) of 2.9%. Three classes of aldehydes, namely 4-hydroxy-2-alkenals, 2-alkenals and 4-hydroxy-2,6-alkedienals―hypothesized to be markers of oxidative stress―were chosen as representative metabolites of interest to evaluate the repeatability and reproducibility of this breath analysis analytical platform. Median and interquartile ranges (IQRs) of CVs for CO_2_, exhalation flow rate, and exhaled volume were 3.2% (1.5%), 3.1% (1.9%), and 5.0% (4.6%), respectively. Despite the high repeatability observed for these parameters, we observed a systematic decay in the signal during repeated measurements for the shorter fatty aldehydes, which eventually reached a steady state after three/four repeated exhalations. In contrast, longer fatty aldehydes showed a steady behavior, independent of the number of repeated exhalation maneuvers. We hypothesize that this highly molecule-specific and individual-independent behavior may be explained by the fact that shorter aldehydes (with higher estimated blood-to-air partition coefficients; approaching 100) mainly get exchanged in the airways of the respiratory system, whereas the longer aldehydes (with smaller estimated blood-to-air partition coefficients; approaching 10) are thought to exchange mostly in the alveoli. Exclusion of the first three exhalations from the analysis led to a median CV (IQR) of 6.7 % (5.5 %) for the said classes of aldehydes. We found that such intra-subject variability is in general much lower than inter-subject variability (median relative differences between subjects 48.2%), suggesting that the system is suitable to capture such differences. No batch effect due to sampling date was observed, overall suggesting that the intra-subject variability measured for these series of aldehydes was biological rather than technical. High correlations found among the series of aldehydes support this notion. Finally, recommendations for breath sampling and analysis for SESI-HRMS users are provided with the aim of harmonizing procedures and improving future inter-laboratory comparisons.

**Electronic supplementary material:**

The online version of this article (10.1007/s00216-019-01764-8) contains supplementary material, which is available to authorized users.

**Figure:**
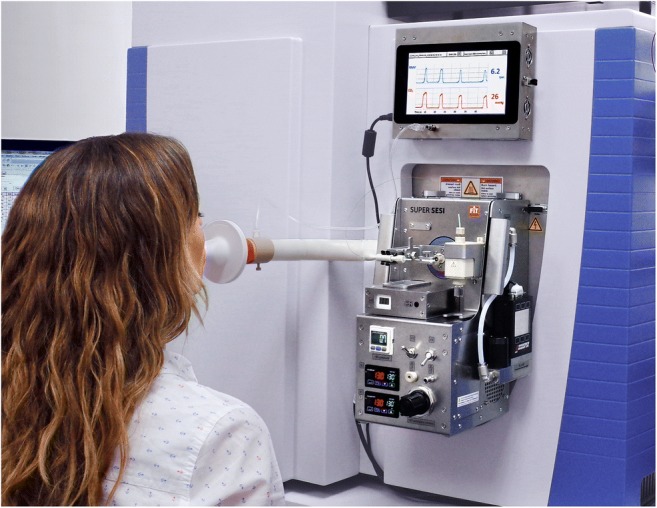
Graphical abstract

## Introduction

Mass spectrometry is a pivotal technique in clinical chemistry laboratories and will continue its expansion to support clinical decision-making [1]. One of such potential future applications is the analysis of exhaled breath metabolites for clinical diagnosis and therapeutic monitoring [2]. However, such an endeavor requires standardized protocols, performed in multi-center studies leading to conclusive evidence, before regulatory authorities can approve a clinical test. In this regard, transitioning from promising research results to concrete clinical applications proves to be a challenge, leading to few routinely used clinical breath tests [3].

A number of analytical techniques have emerged over the last five decades, aiming to address this challenge, being the earliest one gas chromatography-mass spectrometry (GC-MS) [4, 5]. GC-MS and its improved modern variants such as GC×GC-Time of flight remain to be the workhorse platform capable of mapping the yet largely unknown breath metabolome [6]. However, one important limitation of GC-MS is the requirement of sample preparation, which leads to lengthy analyses and poses at the same time additional difficulties to standardize procedures and to preserve chemically uncompromised breath specimens [7]. Since breath constitutes a virtually unlimited source of information, real-time techniques such as proton-transfer-reaction mass spectrometry (PTR-MS) [8] and selected-ion flow-tube mass spectrometry (SIFT-MS) [9] emerged to conveniently capture this information. Such convenient online monitoring of exhaled metabolites is obviously of great advantage. However, it comes at the price of limited sensitivity―as no sample pre-concentration is possible―and limited selectivity―as no chromatographic separation prior to mass analysis is possible. A third real-time mass spectrometric alternative is secondary electrospray ionization-mass spectrometry (SESI-MS) [10]. In contrast to PTR-MS and SIFT-MS, ionization of exhaled metabolites takes place at atmospheric pressure in SESI-MS. The benefit of doing so is twofold: (i) the ionization probability increases with pressure [11] and (ii) it allows to conveniently interface the ionization stage with virtually any pre-existing atmospheric pressure ionization mass analyzer, including ultra-high-resolution (> 100,000) MS such as Orbitrap. This results in sensitive and selective, yet real-time, analysis of trace vapor species. As a result, despite being the most recently proposed mass spectrometric alternative for real-time gas analysis, it is steadily gaining interest across different research groups [10, 12–26]. However, most of the published SESI-MS studies rely on lab-built instrumentation, making it difficult to standardize procedures for this technique. Following ongoing efforts to standardize exhaled breath collection and subsequent analysis for other analytical platforms [27–34], we present here a series of instrumental developments aiming to standardize breath analysis procedures and to provide recommendations for SESI-HRMS users interested in breath analysis. To do so, we characterized a series of new instrumentation with a focus on a panel of three classes of exhaled aldehydes.

## Material and methods

**Fig. 1:**
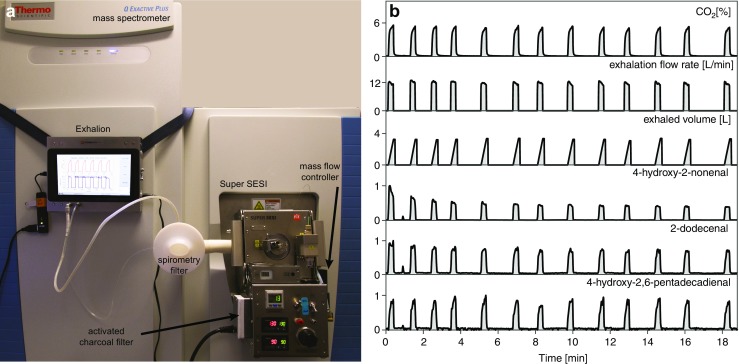
Real-time breath analysis using SESI-HRMS. **a** SESI-HRMS analytical platform located in a clinical setting (University Children’s Hospital Basel) dedicated for real-time breath analysis. The system features three main elements: (i) exhalation interface, which provides feedback to the participants on the exhalation maneuver; (ii) ion source, which efficiently ionizes exhaled metabolites, and (iii) high-resolution mass spectrometer. **b** Real-time analysis by simultaneous monitoring of CO_2_, physical exhalation parameters (exhalation flow rate and exhaled volume), and relative intensities of three representative aldehydes from one experiment. 13 consecutive exhalations within 20 min for one subject are shown (see ESM Fig. S4 for zoomed-in view of the first exhalation).

We investigated the exhaled breath composition of healthy subjects by SESI-HRMS. The breath analysis platform consisted of three main components. The first one was a newly developed interface (Exhalion, FIT, Spain), which measures CO_2_ (%), pressure drop (mbar), exhalation flow rate (L/min), and exhaled volume (L) in real time to guide the exhalation maneuver. Downstream, the exhaled breath is ionized in an ion source (Super SESI, FIT, Spain). Ionized breath metabolites were then analyzed in real time by a high-resolution mass spectrometer (Q Exactive Plus, Thermo Fisher Scientific, Germany). Figure 1 a shows a picture of the breath analysis platform.

### Exhalation maneuver monitoring and guiding (Exhalion)

The breath interface Exhalion was constructed with the aim of assisting in the control and reproducibility of exhalation maneuver. Exhalion consists of the following elements: a disposable standard antibacterial/antiviral medical grade filter. In this study, commercially available spirometry filters (MicroGard™, Vyaire Medical, USA; 3 cm ID; filters 99.98% of bacteria and 99.92% of viruses) were used as a mouthpiece. Downstream, the filter is connected to an autoclavable interface, housing a calibrated flow restriction. By measuring the pressure drop through the calibrated restriction (range 0 to 20 mbar, accuracy 2.5%, precision 0.1 mbar), Exhalion determines the flow rate (range 0 to 15 L/min, accuracy 2.5%), and total exhaled volume (the latter is automatically estimated by detecting the onset of the exhalation and integrating flow rate over time). Capnography data is measured side-stream (range 0 to 20%, accuracy 5% of the reading), with an approximate flow rate of 0.5 L/min. Absolute pressure measurement is also integrated and is used to compensate for the effect of barometric variations on CO_2_ and flow readings. Time and other parameters are measured at a rate of 1.5 Hz, and stored in a text file. Finally, a main module, incorporating a touch screen, a micro-computer, all sensors, and a dedicated firmware to run autonomously, is used to process all the data from flow restriction and capnograph in real time. All routines to seamlessly calibrate the sensors are integrated into the firmware. The main module and the flow restriction interface are connected with two tubes (1/8” OD, for CO_2_ and pressure measurement). Nafion tubing was used to prevent condensation. The dead volume of the side-stream tubing and the sensors was below 5 cm^3^, which provides an upper limit for the CO_2_ reading delay of 0.5 s. The total dead volume was dominated by the mouthpiece filter, as Exhalion was designed to minimize this contribution. The Exhalion device was connected downstream with the ionization device (Super SESI).

### Secondary electrospray ionization (Super SESI)

The Super SESI source was optimized for breath analysis and integrates all components required to control the ionization of the sample flow. A fraction of the total exhaled flow is passed to the ionizer, which features a sampling line connected to an ionization chamber whereby a nano-electrospray (0.1% ammonium formate in water) ionizes the metabolites present in breath. We used a 20-μm ID TaperTip (New Objective, USA) silica capillary emitter. The Super SESI pressure was set to 1.3 bar to drive the liquid through the capillary. The steady-state reading of the nano-amperemeter indicated that a stable spray was formed (typically 130 nA). The sampling line temperature was set to 130 °C and the ion chamber temperature was set to 90 °C. In addition, the sampling line and the ionization chamber core were silica-coated to minimize analyte adsorption onto the system walls. Super SESI uses a flow of clean nitrogen (filtered through a built-in activated charcoal filter) to sweep the ionizer when there is no sample input. It was set to provide an excess of 0.4 L/min over the flow ingested by the mass spectrometer (precise reading and control of this is integrated into the Super SESI). The exhaust mass flow controller was then set to 0.7 L/min so that the fraction of breath entering the ionizer was fixed at 0.3 L/min regardless of potential exhalation pressure fluctuations. The dead volume of the sample line and the ionizer was approximately 10 cm^3^. At this flow, the time required for breath to reach and sweep the ionizer is 2 s.

### High-resolution mass spectrometry (Q Exactive Plus)

The Super SESI source was directly coupled to the Q Exactive Plus MS and was recognized as an ESI source (sheath gas flow rate 60, auxiliary gas flow rate 2, spray voltage 3.5 kV, capillary temperature 275 °C, and S-lens RF level 55.0). The MS was operated directly via Q Exactive Tune software (version 2.9) in full MS mode (polarity positive, scan range 100 to 400 *m*/*z*, microscans 4, ACG target 10^6^, and maximum injection time 500 ms) with a resolution of 140,000 (at *m/z* 200). The MS was externally calibrated on a weekly basis using a commercially available calibration solution (Pierce™ Triple Quadrupole, extended mass range) and internally calibrated by enabling lock masses (*m/z* 149.02332, 279.15909, 355.06993, 371.10123, and 391.28429), which correspond to common background mass spectrometric contaminants [35, 36].

### Subjects

Three male and one female healthy subjects (33 ± 8 years, mean ± SD) were enrolled in the study, each subject provided at least 49 exhalations. All measurements were performed during weekdays at any given time between 8 a.m. and 6 p.m. Fig. S1 (see Electronic Supplementary Material, ESM) shows the measurement scheduling distribution for all participants, indicating no significant bias towards a specific time window for any given subject. The sample size and number of replicates resulted from estimating the within-subject standard deviation, following the approach described by Bland and Altman [37]. Shortly, the precision with which one can estimate within-subject standard deviation depends on both the number of subjects and the number of observations per subject. Details are described in the ESM (Table S1). The subjects provided prolonged exhalations, whereby the subjects inspired to total lung capacity and expired at a constant flow rate. This expiration maneuver was repeated at least six consecutive times with breaks of at least 10 s in-between replicate exhalations. Typically, the total exhaled volume per exhalation was 3 L. To guide the maneuver, the subjects could monitor in real time their CO_2_ level, exhalation flow rate, and exhaled volume on the Exhalion touch screen. Fig. S2 (see ESM) shows a picture of how a subject would perform the breath test.

### Data analysis

Raw data from the MS and Exhalion device were exported and processed using MATLAB (version 2018a, MathWorks Inc., USA). Briefly, raw MS data were converted into mzXML file format using ProteoWizard’s msConvertGUI [38]. Afterwards, each spectrum from all files was aligned and calibrated using the RAFFT algorithm implemented in MATLAB [39]. Then *mspeaks* and *ksdensity* functions of MATLAB were used to appropriately pick and extract the final feature list of 2,255 features. Molecular formulae were generated based on the accurate mass by considering C, H, N, and O [40]. A number of studies suggest using CO_2_- and volume-controlled sampling maneuvers as a standardization procedure [34, 41–44]. Following the recommendations to use this physiological parameter to normalize breath analysis data, we normalized signal intensities by considering exhalation windows where the CO_2_ concentrations rose above 3%. In particular, we computed the signal intensity for each of these 2,255 features during each exhalation by using the time corresponding to 3% or more of the CO_2_ signal (from Exhalion) to define a single exhalation event. Finally, the integrated area under the curve (AUC) for all features was normalized by the exhaled volume in the exhalation (calculated by integrating the flow over time in a particular exhalation from Exhalion data). We will henceforth refer to this normalized AUC as breath-signal.

**Fig. 2:**
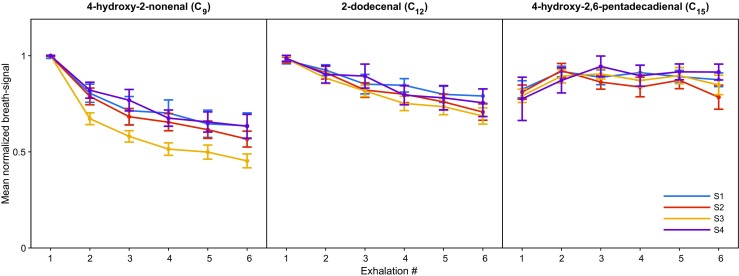
Aldehydes show a subject-independent and molecule-dependent exhalation pattern. Data shown is the mean normalized breath-signal with errors bars representing 95% CIs for three selected aldehydes from four subjects (denoted as S1–S4) in 104 experiments as a function of exhalation number. Lighter species show a systematic decaying trend across consecutive exhalations, which is subject independent.

For Fig. 2 and Figs. S6-S8 (see ESM), we first normalized the breath-signal of metabolites from each experiment (containing 6 exhalations) to the maximum. Then, normalized breath-signals of metabolites were averaged across different experiments to obtain the final “mean normalized breath-signal” of metabolites for each subject along with their 95% confidence interval (CI).

Intra-subject variability for each feature was estimated by calculating the coefficient of variation (CV, expressed as percentage) of the replicate exhalations (this analysis led to Table 1). Inter-subject variability was evaluated by performing one-way analysis of variance (ANOVA; grouped by subjects), followed by a multiple comparison (post hoc) test, using the Bonferroni method, to determine whether pairs of group means were significantly different (this analysis led to Table 2).

**Table 1:** Intra-subject variability in the breath-signal for the series of aldehydes studied in this work. The median and IQR values of the CVs (expressed as percentage) measured for the four subjects for the 27 aldehydes studied are listed; *DBE* double bond equivalent

*m/z* ([M + H]^+^)	Metabolite				Coefficient of variation (%)							
					S1 (*N* = 25)		S2 (*N* = 29)		S3 (*N* = 37)		S4 (*N* = 13)	
	Formula (M)	Name	DBE	Mass error (ppm)	Median	IQR	Median	IQR	Median	IQR	Median	IQR
143.1066	C8H14O2	4-Hydroxy-2-octenal	2	− 0.32	6.2	6.7	7.5	4.2	6.3	6.0	4.3	2.7
157.1223	C9H16O2	4-Hydroxy-2-nonenal	2	0.16	6.8	6.0	8.7	4.6	7.8	7.4	4.9	2.4
171.1379	C10H18O2	4-Hydroxy-2-decenal	2	− 0.15	7.3	5.3	7.5	4.8	7.3	5.8	4.3	2.2
185.1537	C11H20O2	4-Hydroxy-2-undecenal	2	0.30	6.8	4.8	7.5	4.2	6.9	6.5	4.4	3.8
199.1694	C12H22O2	4-Hydroxy-2-dodecenal	2	0.58	7.6	8.5	8.0	4.7	7.7	6.6	4.6	3.6
213.1851	C13H24O2	4-Hydroxy-2-tridecenal	2	0.68	7.6	6.7	7.9	4.7	6.5	5.5	4.7	2.5
227.2005	C14H26O2	4-Hydroxy-2-tetradecenal	2	− 0.11	5.6	4.5	7.2	3.9	6.6	5.6	5.5	4.0
241.2162	C15H28O2	4-Hydroxy-2-pentadecenal	2	− 0.11	4.8	5.1	6.1	5.0	6.1	3.8	5.7	3.2
255.2319	C16H30O2	4-Hydroxy-2-hexadecenal	2	− 0.02	6.2	5.0	5.1	5.0	6.0	5.8	7.1	3.4
127.1118	C8H14O	2-Octenal	2	0.07	6.5	3.8	6.3	4.2	7.3	8.5	7.4	6.5
141.1275	C9H16O	2-Nonenal	2	0.42	7.4	5.2	7.5	3.4	7.1	6.3	5.8	3.9
155.1429	C10H18O	2-Decenal	2	− 0.66	6.0	7.0	6.6	5.4	6.0	5.9	4.0	4.7
169.1587	C11H20O	2-Undecenal	2	− 0.01	6.6	7.0	8.1	4.2	7.4	5.6	5.7	5.3
183.1744	C12H22O	2-Dodecenal	2	0.49	6.7	4.9	7.7	5.2	6.9	6.3	5.4	2.2
197.1901	C13H24O	2-Tridecenal	2	0.56	6.5	7.3	7.0	3.5	7.5	7.0	5.5	4.6
211.2055	C14H26O	2-tetradecenal	2	− 0.53	7.5	5.7	7.8	4.9	6.8	5.9	7.1	4.9
225.2213	C15H28O	2-Pentadecenal	2	− 0.09	7.6	7.7	7.8	4.1	7.4	5.2	6.8	5.0
239.2369	C16H30O	2-Hexadecenal	2	− 0.05	8.3	7.0	12.8	12.2	8.4	7.2	8.0	8.4
141.0910	C8H12O2	4-Hydroxy-2,6-octadienal	3	0.25	5.4	5.8	7.3	3.6	5.7	4.8	4.5	3.0
155.1066	C9H14O2	4-Hydroxy-2,6-nonadienal	3	− 0.62	5.4	7.6	7.7	3.8	6.7	6.5	5.3	2.8
169.1223	C10H16O2	4-Hydroxy-2,6-dodecadienal	3	0.09	7.4	6.9	7.9	4.5	5.8	5.1	5.4	3.8
183.1380	C11H18O2	4-Hydroxy-2,6-undecadienal	3	0.46	7.1	6.3	7.8	3.5	7.7	5.7	4.5	3.0
197.1537	C12H20O2	4-Hydroxy-2,6-dodecadienal	3	0.58	7.6	5.5	7.7	4.5	5.3	4.3	4.8	3.0
211.1692	C13H22O2	4-Hydroxy-2,6-tridecadienal	3	− 0.41	7.0	7.3	7.7	5.0	6.0	5.6	5.1	4.3
225.1849	C14H24O2	4-Hydroxy-2,6-tetradecadienal	3	− 0.07	6.6	6.5	6.6	5.3	6.0	6.2	5.1	3.4
239.2005	C15H26O2	4-Hydroxy-2,6-pentadecadienal	3	− 0.11	7.4	6.6	11.7	13.5	5.8	4.7	5.3	6.2
253.2162	C16H28O2	4-Hydroxy-2,6-hexadecadienal	3	− 0.02	8.1	9.2	4.6	4.4	6.3	5.5	5.6	3.8

**Table 2:** Pairwise inter-subject variability in the breath-signal for the series of aldehydes studied in this work. Table shows the relative differences in aldehyde breath-signals between all subject pairings together with the lower and upper bounds (LB and UB) of the 95% CI and *p*-values

*m/z *([M + H]^+^)	Metabolite				S1 vs S2				S1 vs S3				S1 vs S4			
	Formula (M)	Name	DBE	Mass error (ppm)	% change	LB	UB	*p* value	% change	LB	UB	*p* value	% change	LB	UB	*p* value
143.1066	C_8_H_14_O_2_	4-Hydroxy-2-Octenal	2	− 0.32	− 30.1	−54.6	− 5.6	7.98 *×* 10^−3^	− 10.4	− 33.6	12.9	1	− 3.0	− 33.7	27.7	1
157.1223	C_9_H_16_O_2_	4-Hydroxy-2-Nonenal	2	0.16	− 42.4	− 67.9	− 17.0	1.17 *×* 10^−4^	− 6.6	− 30.7	17.6	1	− 14.2	− 46.1	17.7	1
171.1379	C_10_H_18_O_2_	4-Hydroxy-2-Decenal	2	− 0.15	− 22.5	− 56.0	11.0	4.41 *×* 10^−1^	− 23.0	− 54.7	8.8	3.28 *×* 10^−1^	18.6	− 23.4	60.6	1
185.1537	C_11_H_20_O_2_	4-Hydroxy-2-Undecenal	2	0.30	− 56.4	− 96.0	− 16.9	1.28 *×* 10^−3^	3.0	− 34.5	40.5	1	− 69.7	− 119.2	− 20.2	1.54 *×* 10^−3^
199.1694	C_12_H_22_O_2_	4-Hydroxy-2-Dodecenal	2	0.58	− 30.4	− 56.6	− 4.3	1.37 *×* 10^−2^	− 4.2	− 29.0	20.6	1	− 11.5	− 44.3	21.3	1
213.1851	C_13_H_24_O_2_	4-Hydroxy-2-Tridecenal	2	0.68	− 33.8	− 56.7	− 10.8	8.40 *×* 10^−4^	− 1.2	− 23.0	20.6	1	− 18.5	− 47.2	10.3	5.22 *×* 10^−1^
227.2005	C_14_H_26_O_2_	4-Hydroxy-2-Tetradecenal	2	− 0.11	− 60.0	− 88.5	− 31.6	7.74 *×* 10^−7^	1.6	− 25.4	28.6	1	− 84.0	− 119.6	− 48.4	3.89 *×* 10^−8^
241.2162	C_15_H_28_O_2_	4-Hydroxy-2-Pentadecenal	2	− 0.11	− 44.0	− 67.6	− 20.4	1.35 *×* 10^−5^	2.9	− 19.5	25.3	1	− 38.2	− 67.8	− 8.7	4.44 *×* 10^−3^
255.2319	C_16_H_30_O_2_	4-Hydroxy-2-Hexadecenal	2	− 0.02	− 36.2	− 62.9	− 9.5	2.53 *×* 10^−3^	10.8	− 14.5	36.1	1	− 5.3	− 38.7	28.2	1
127.1118	C_8_H_14_O	2-Octenal	2	0.07	− 73.7	− 125.0	− 22.4	1.18 *×* 10^−3^	4.9	− 43.8	53.5	1	− 56.5	− 120.7	7.8	1.19 *×* 10^−1^
141.1275	C_9_H_16_O	2-Nonenal	2	0.42	− 82.7	− 114.8	− 50.7	2.34 *×* 10^−9^	14.8	− 15.6	45.3	1	− 100.8	− 141.0	− 60.7	5.73 *×* 10^−9^
155.1429	C_10_H_18_O	2-Decenal	2	− 0.66	20.1	− 11.8	52.1	5.56 *×* 10^−1^	89.3	59.0	119.6	1.85 *×* 10^−11^	78.7	38.6	118.7	4.30 *×* 10^−6^
169.1587	C_11_H_20_O	2-Undecenal	2	− 0.01	− 88.0	− 144.0	− 31.9	3.16 *×* 10^−4^	5.1	− 48.0	58.3	1	− 139.7	− 209.9	− 69.5	3.28 *×* 10^−6^
183.1744	C_12_H_22_O	2-Dodecenal	2	0.49	− 57.9	− 95.8	− 20.0	4.83 *×* 10^−4^	9.8	− 26.1	45.8	1	− 99.4	− 146.8	− 51.9	9.72 *×* 10^−7^
197.1901	C_13_H_24_O	2-Tridecenal	2	0.56	− 39.3	− 68.0	− 10.7	2.16 *×* 10^−3^	11.9	− 15.3	39.1	1	− 27.4	− 63.4	8.5	2.54 *×* 10^−1^
211.2055	C_14_H_26_O	2-Tetradecenal	2	− 0.53	− 50.3	− 78.3	− 22.3	2.88 *×* 10^−5^	− 5.2	− 31.8	21.4	1	− 78.0	− 113.1	− 42.9	2.06 *×* 10^−7^
225.2213	C_15_H_28_O	2-Pentadecenal	2	− 0.09	− 47.7	− 81.0	− 14.5	1.19 *×* 10^−3^	− 13.1	− 44.7	18.4	1	− 60.3	− 102.0	− 18.7	1.06 *×* 10^−3^
239.2369	C_16_H_30_O	2-Hexadecenal	2	− 0.05	− 35.5	− 67.6	− 3.4	2.19 *×* 10^−2^	− 3.6	− 34.1	26.8	1	− 6.1	− 46.3	34.1	1
141.0910	C_8_H_12_O_2_	4-Hydroxy-2,6-Octadienal	3	0.25	− 39.9	− 65.2	− 14.6	2.96 *×* 10^−4^	9.9	− 14.1	33.9	1	− 23.6	− 55.3	8.1	2.88 *×* 10^−1^
155.1066	C_9_H_14_O_2_	4-Hydroxy-2,6-Nonadienal	3	− 0.62	− 54.8	− 83.4	− 26.3	7.14 *×* 10^−6^	7.0	− 20.1	34.0	1	− 43.6	− 79.3	− 7.8	8.54 *×* 10^−3^
169.1223	C_10_H_16_O_2_	4-Hydroxy-2,6-Dodecadienal	3	0.09	− 72.4	− 99.8	− 45.0	1.06 *×* 10^−9^	− 10.0	− 36.0	16.0	1	− 24.3	− 58.6	10.0	3.58 *×* 10^−1^
183.1380	C_11_H_18_O_2_	4-Hydroxy-2,6-Undecadienal	3	0.46	− 95.4	− 179.2	− 11.7	1.66 *×* 10^−2^	1.3	− 78.1	80.7	1	− 166.9	− 271.8	− 62.0	2.55 *×* 10^−4^
197.1537	C_12_H_20_O_2_	4-Hydroxy-2,6-Dodecadienal	3	0.58	− 50.3	− 91.0	− 9.6	7.41 *×* 10^−3^	− 20.8	− 59.4	17.8	9.01 *×* 10^−1^	− 74.6	− 125.6	− 23.7	9.04 *×* 10^−4^
211.1692	C_13_H_22_O_2_	4-Hydroxy-2,6-Tridecadienal	3	− 0.41	− 51.6	− 76.1	− 27.2	7.93 *×* 10^−7^	− 5.5	− 28.7	17.7	1	− 37.4	− 68.0	− 6.7	8.53 10^−3^
225.1849	C_14_H_24_O_2_	4-Hydroxy-2,6-Tetradecadienal	3	− 0.07	− 74.0	− 108.8	− 39.1	6.82 *×* 10^−7^	3.3	− 29.7	36.4	1	− 90.0	− 133.7	− 46.3	1.42 *×* 10^−6^
239.2005	C_15_H_26_O_2_	4-Hydroxy-2,6-Pentadecadienal	3	− 0.11	− 31.3	− 54.1	− 8.4	2.22 *×* 10^−3^	6.4	− 15.2	28.1	1	− 55.5	− 84.1	− 26.8	6.02 *×* 10^−6^
253.2162	C_16_H_28_O_2_	4-Hydroxy-2,6-Hexadecadienal	3	− 0.02	− 24.0	− 47.1	− 1.0	3.61 *×* 10^−2^	8.7	− 13.1	30.6	1	− 21.0	− 49.9	7.8	3.16 *×* 10^−1^

					S1 vs S2				S1 vs S3				S1 vs S4			
					% change	LB	UB	*p* value	% change	LB	UB	*p* value	% change	LB	UB	*p* value
143.1066	C_8_H_14_O_2_	4-Hydroxy-2-Octenal	2	− 0.32	15.1	− 2.0	32.3	1.15 *×* 10^−1^	20.8	− 2.3	43.8	1.02 *×* 10^−1^	6.7	− 19.6	32.9	1
157.1223	C_9_H_16_O_2_	4-Hydroxy-2-Nonenal	2	0.16	25.2	8.9	41.4	3.88 *×* 10^−4^	19.8	− 2.1	41.7	9.96 *×* 10^−2^	− 7.2	− 35.4	21.0	1
171.1379	C_10_H_18_O_2_	4-Hydroxy-2-Decenal	2	− 0.15	− 0.4	− 25.2	24.5	1	33.5	0.1	67.0	4.89 *×* 10^−2^	33.8	1.6	66.0	3.43 *×* 10^−2^
185.1537	C_11_H_20_O_2_	4-Hydroxy-2-Undecenal	2	0.30	38.0	15.0	60.9	1.33 *×* 10^−4^	− 8.5	− 39.4	22.4	1	− 74.9	− 123.1	− 26.8	3.59 *×* 10^−4^
199.1694	C_12_H_22_O_2_	4-Hydroxy-2-dodecenal	2	0.58	20.1	1.9	38.3	2.25 *×* 10^−2^	14.5	− 10.0	39.0	6.84 *×* 10^−1^	− 7.0	− 36.6	22.7	1
213.1851	C_13_H_24_O_2_	4-Hydroxy-2-Tridecenal	2	0.68	24.4	8.8	39.9	3.41 *×* 10^−4^	11.4	− 9.5	32.4	8.72 *×* 10^−1^	− 17.1	− 43.9	9.7	5.36 *×* 10^−1^
227.2005	C_14_H_26_O_2_	4-Hydroxy-2-Tetradecenal	2	− 0.11	38.5	22.4	54.7	2.70 *×* 10^−8^	− 14.9	− 36.7	6.8	4.02 *×* 10^−1^	− 87.0	− 121.1	− 52.9	3.49 *×* 10^−9^
241.2162	C_15_H_28_O_2_	4-Hydroxy-2-Pentadecenal	2	− 0.11	32.6	17.7	47.4	3.17 *×* 10^−7^	4.0	− 16.0	24.0	1	− 42.3	− 71.0	− 13.6	8.10 *×* 10^−4^
255.2319	C_16_H_30_O_2_	4-Hydroxy-2-Hexadecenal	2	− 0.02	34.5	16.7	52.3	6.02 *×* 10^−6^	22.7	− 1.3	46.7	7.39 *×* 10^−2^	− 18.0	− 53.4	17.3	1
127.1118	C_8_H_14_O	2-Octenal	2	0.07	45.2	18.4	72.1	9.54 *×* 10^−5^	9.9	− 26.2	46.0	1	− 64.5	− 128.2	− 0.8	4.54 *×* 10^−2^
141.1275	C_9_H_16_O	2-Nonenal	2	0.42	53.4	37.4	69.3	8.66 *×* 10^−14^	− 9.9	− 31.4	11.5	1	− 135.8	− 180.3	− 91.4	4.58 *×* 10^−12^
155.1429	C_10_H_18_O	2-Decenal	2	− 0.66	86.7	50.3	123.0	2.79 *×* 10^−8^	73.3	24.4	122.2	6.47 *×* 10^−4^	− 100.2	− 454.2	253.7	1
169.1587	C_11_H_20_O	2-Undecenal	2	− 0.01	49.5	22.4	76.6	2.03 *×* 10^−5^	− 27.5	− 64.0	8.9	2.69 *×* 10^−1^	− 152.7	− 222.5	− 82.9	3.14 *×* 10^−7^
183.1744	C_12_H_22_O	2-Dodecenal	2	0.49	42.9	21.1	64.7	4.29 *×* 10^−6^	− 26.3	− 55.6	3.1	1.07 *×* 10^−1^	− 121.1	− 170.7	− 71.4	1.39 *×* 10^−8^
197.1901	C_13_H_24_O	2-Tridecenal	2	0.56	36.8	18.1	55.5	4.21 *×* 10^−6^	8.5	− 16.6	33.7	1	− 44.7	− 83.1	− 6.2	1.38 *×* 10^−2^
211.2055	C_14_H_26_O	2-Tetradecenal	2	− 0.53	30.0	13.1	46.9	3.76 *×* 10^−5^	− 18.4	− 41.2	4.4	1.92 *×* 10^−1^	− 69.2	− 100.6	− 37.7	2.71 *×* 10^−7^
225.2213	C_15_H_28_O	2-Pentadecenal	2	− 0.09	23.4	3.0	43.9	1.59 *×* 10^−2^	− 8.5	− 36.1	19.0	1	− 41.7	− 76.5	− 7.0	9.93 *×* 10^−3^
239.2369	C_16_H_30_O	2-Hexadecenal	2	− 0.05	23.5	2.0	45.0	2.45 *×* 10^−2^	21.7	− 7.3	50.7	2.80 *×* 10^−1^	− 2.4	− 39.0	34.2	1
141.0910	C_8_H_12_O_2_	4-Hydroxy-2,6-Octadienal	3	0.25	35.6	19.2	52.1	4.11 *×* 10^−7^	11.7	− 10.5	33.8	9.56 *×* 10^−1^	− 37.2	− 70.4	− 4.0	1.95 *×* 10^−2^
155.1066	C_9_H_14_O_2_	4-Hydroxy-2,6-Nonadienal	3	− 0.62	39.9	23.2	56.6	2.84 *×* 10^−8^	7.3	− 15.3	29.8	1	− 54.3	− 90.5	− 18.1	6.40 *×* 10^−4^
169.1223	C_10_H_16_O_2_	4-Hydroxy-2,6-Dodecadienal	3	0.09	36.2	21.7	50.6	6.08 *×* 10^−9^	27.9	8.5	47.3	1.20 *×* 10^−3^	− 13.0	− 42.4	16.4	1
183.1380	C_11_H_18_O_2_	4-Hydroxy-2,6-Undecadienal	3	0.46	49.5	10.6	88.4	5.38 *×* 10^−3^	− 36.6	− 88.9	15.8	3.80 *×* 10^−1^	− 170.5	− 270.7	− 70.2	8.13 *×* 10^−5^
197.1537	C_12_H_20_O_2_	4-Hydroxy-2,6-Dodecadienal	3	0.58	19.6	− 5.0	44.2	2.06 *×* 10^−1^	− 16.2	− 49.3	16.9	1	− 44.6	− 84.4	− 4.8	1.96 *×* 10^−2^
211.1692	C_13_H_22_O_2_	4-Hydroxy-2,6-Tridecadienal	3	− 0.41	30.4	15.8	45.1	1.18 *×* 10^−6^	9.4	− 10.3	29.1	1	− 30.3	− 57.7	− 2.9	2.22 *×* 10^−2^
225.1849	C_14_H_24_O_2_	4-Hydroxy-2,6-Tetradecadienal	3	− 0.07	44.4	26.2	62.6	1.36 *×* 10^−8^	− 9.2	− 33.7	15.3	1	− 96.6	− 139.1	− 54.0	1.20 *×* 10^−7^
239.2005	C_15_H_26_O_2_	4-Hydroxy-2,6-Pentadecadienal	3	− 0.11	28.7	12.9	44.6	2.31 *×* 10_−5_	− 18.4	− 39.7	2.9	1.32 *×* 10^−1^	− 66.2	− 95.0	− 37.3	8.76 *×* 10^−8^
253.2162	C_16_H_28_O_2_	4-Hydroxy-2,6-Hexadecadienal	3	− 0.02	26.4	9.5	43.3	3.35 *×* 10^−4^	2.4	− 20.3	25.2	1	− 32.6	− 62.5	− 2.8	2.43 *×* 10^−2^

### Gas standard generation (ReGaS2)

To monitor the stability of the ionization, a reactive gas standard generator (ReGaS2) developed by the Swiss Federal Institute of Metrology (METAS) [45], was used. This device releases a flow with stable concentrations of trace gases and can be used to standardize gas sensors. In our case, β-pinene at a concentration of 92.7 ppb in air was used as target vapor (carrier flow of 1 L/min and dilution flow of 0.5 L/min at an oven temperature of 41 °C).

## Results and discussion

### Technical variability measured using β-pinene vapors

Before discussing the biological variability measured in human breath, we gauged the typical technical variability to be expected for our SESI-HRMS system. In order to do so, we infused a continuous stream of air seeded with 92.7 ppb of β-pinene, simulating an exhalation maneuver. Upon injection of the standard, the mass spectrum was dominated by the expected protonated β-pinene at *m/z* 137.1326 (C_10_H_17_), along with some oxidized species (C_10_H_15_O and C_10_H_17_O_2_; ESM Fig. S3). SESI-MS is known to detect trace species down to the sub-ppt range [22]. For this reason, and not surprisingly, 92.7 ppb of β-pinene nearly saturated the detector of the Orbitrap mass analyzer. Because the dynamic range of our mass analyzer is five orders of magnitude (signal intensity 10^4^–10^9^ a.u.), the limit of detection is expected to be at around 1 ppt, which is consistent with previous SESI-MS quantification studies [46]. When we started the delivery of β-pinene, the signal of the protonated analyte raised sharply to reach a plateau. We measured the stability of the signal intensity detection during 1 h. When the delivery of β-pinene was stopped, the signal intensity dropped abruptly to baseline level, indicating no carryover effects, at least for this particular compound (inset Fig. S3, see ESM). The CV of β-pinene signal intensity during an hour of continuous delivery of the vapor was found to be 2.3%. We therefore conclude that technical CVs within 3% are to be expected for our SESI-HRMS platform.

### Replicate exhalations: intra- and inter-subject variability

In total, the four participants provided 648 exhalations (*n* = 171 for subject 1, *n* = 174 for subject 2, *n* = 225 for subject 3 and *n* = 78 for subject 4). These measurements were subdivided into 104 single experiments (*N* = 25 for subject 1, *N* = 29 for subject 2, *N* = 37 for subject 3, and *N* = 13 for subject 4) each containing 6 to 13 exhalations (replicates) performed within 10 to 20 min. The aim was to examine the variability across these replicates, considering that the technical variability, as mentioned above, was found to be in the range of 3%. Figure 1 b shows one such representative experiment whereby a subject provided 13 consecutive exhalations during 19 min (ESM Fig. S4 shows a zoomed-in view of the first exhalation, where the time traces can be inspected in greater detail).

The vast majority of the features typically detected by SESI-HRMS in human breath remain to be positively identified. However, over the last years, we have made a substantial effort to systematically identify the molecular structure for some of these metabolites by combining real-time breath MS/MS analysis and UPLC-MS/MS analysis of exhaled breath condensate [47–53]. Given the clinical importance of aldehydes, as potential surrogates of oxidative stress [54–59], we will concentrate in discussing our findings for a series of three classes of fatty aldehydes [48]: 4-hydroxy-2-alkenals (C_*x*_H_2*x* – 2_O_2_), 2-alkenals (C_*x*_H_2*x* − 2_O), and 4-hydroxy-2,6-alkadienals (C_*x*_H_2*x* − 4_O_2_) with chain lengths ranging from C_8_ to C_16_. These 27 representative aldehydes were used as benchmarking metabolites. For reference, Fig. 1 b shows the time traces of three such representative exhaled aldehydes and Fig. S5 (see ESM) shows the time traces for the 27 aldehydes of interest from the same experiment. The gray areas in Fig. 1 b and Fig. S4 (see ESM) represent the time windows whereby CO_2_ levels were above 3%.

Visual inspection of CO_2_ and exhalation parameters from Fig. 1b suggests a high repeatability across replicate measurements. Indeed, computed mean ± SD for this particular experiment yielded a CO_2_ level of 4.7 ± 0.1%, an exhalation flow rate of 11.7 ± 0.3 L/min and an exhaled volume of 2.6 ± 0.1 L (i.e., excluding 0.5–0.6 L of breath not containing at least 3% of CO_2_) for the considered windows. Median CVs (IQRs) for CO_2_, exhalation flow rate, and exhaled volume based on all 104 experiments were 3.2% (1.5%), 3.1% (1.9%), and 5.0% (4.6%), respectively. The overall picture for the aldehydes was somehow more complex. While 4-hydroxy-2,6-pentadecadienal in Fig. 1 b shows a relatively constant behavior across all exhalations (akin to CO_2_), 2-dodecenal drops over time during consecutive exhalations and the decay is even more pronounced for 4-hydroxy-2-nonenal, whose signal intensity decays by ~ 35% during the first three exhalations, to then reach a steady state. Interestingly, we observed this behavior systematically for these particular molecules among all participants. Figure 2 shows the mean normalized breath-signal (see “Material and methods” for details) and the corresponding 95% CI from all experiments for the four participants as a function of exhalation number for the three selected representative compounds shown in Fig. 1 b. It clearly shows that the dynamics for each compound are subject independent and, interestingly, seem to depend on the aldehyde chain length. For example, signal intensity drops between the first and the sixth exhalation for 4-hydroxy-2-nonenal is around 50%, for 2-dodecenal the drop is around 30%, whereas for 4-hydroxy-2,6-pentadecadienal signal intensity remains stable (or even increases after the first exhalation). This trend was systematically observed for all the aldehydes from the three classes (ESM Figs. S6-S8)**.**

### Location within the respiratory system where the gas exchange occurs may explain the molecule-dependent exhalation traces

**Fig. 3:**
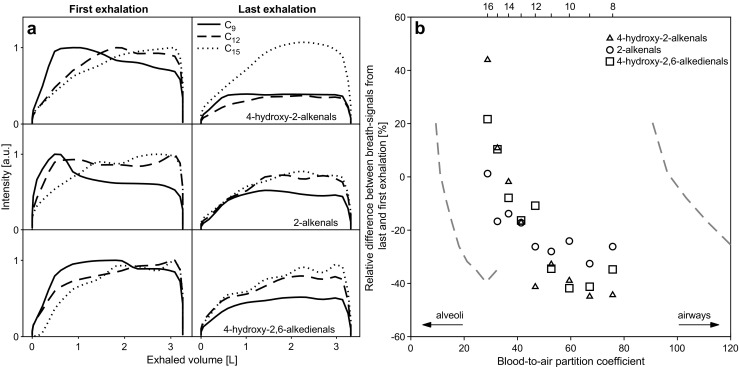
Dependency of exhalation profile of breath metabolites with blood-to-air partition coefficient. **a** Exhalation profiles of short and long aldehydes as a function of exhaled volume is consistent with the hypothesis that the shorter aldehydes exchange mostly in the airways, while longer aldehydes exchange in the alveoli. 4-hydroxy-2-dodecenal shows a deviating pattern that may be caused by an interfering peak. **b** Relative difference between breath-signals from last and first exhalation as a function of predicted blood-to-air partition coefficient. A number of carbon atoms for molecules are shown at the top and gray dashed curves shows the 95% CI from *λ*_*b:a*_ estimation. A large partition coefficient is associated with a strongly decaying pattern (also see ESM Fig. S10, with *x*-axis on log_10_ scale, showing the complete range for 95% CIs)

The signal intensity decaying behavior as a function of chain length can be rationalized by the dependency with Ostwald blood-air partition coefficient (*λ*_*b:a*_), which is the most important factor in determining the location within the respiratory system where the gas exchange occurs [60]. Soluble gases with *λ*_*b:a*_ > 100 exchange almost exclusively within the airways (with the bronchial blood), whereas those with 10 < *λ*_*b:a*_ < 100 exchange partially in the airways and in the alveoli, and those with a *λ*_*b:a*_ < 10 nearly exclusively exchange in the alveoli (with the pulmonary blood) [61]. Therefore, CO_2_ (*λ*_*b:a*_ = 3) exchanges in the alveoli [62]. Figure 1 b shows that the CO_2_ level does not decrease as the participant provides consecutive exhalations and this was the trend observed across all measurements. The same trend is observed for the longest aldehydes, which in turn have the lowest *λ*_*b:a*_ from the series. The predicted *λ*_*b:a*_ by Kramer et al. [63] suggests that, indeed, shorter aldehydes have a greater *λ*_*b:a*_. For example, the predicted *λ*_*b:a*_ for 2-hexenal was 111, therefore exchanges almost exclusively in the airways. In contrast, 2-undecenal has a predicted *λ*_*b:a*_ = 39; hence, it exchanges partially in the airways and in the alveoli. It is expected that even longer aldehydes (> C_14_), such as those studied in this work, will have a *λ*_*b:a*_ approaching the critical value of 10 (i.e., almost exclusively exchanged in the alveoli). This trend can be observed in Fig. S9 (see ESM), which shows predicted *λ*_*b:a*_ as a function of the number of carbon atoms from the aldehydes, based on data by Kramer et al. [63]. Thus, we hypothesize that the longest aldehydes studied here (C_14_–C_16_) exchange exclusively in the alveoli, and for this reason show a similar behavior as CO_2_. In contrast, the smaller aldehydes exchange mainly in the airways, leading to a decrease during prolonged consecutive exhalations. For example, it has been estimated that ethanol, which has a high blood solubility (*λ*_*b:a*_ = 1,803), can show a 20% lower concentration than alveolar air after a complete prolonged exhalation [62]. Reinforcing this idea, we found that the signal intensity as a function of exhaled volume during a single exhalation, varies significantly depending on the aldehyde chain length and therefore on their *λ*_*b:a*_. Figure 3 a displays signal intensity profiles of the aldehydes as a function of exhaled volume for a representative first and last exhalation in an experiment (same experiment as Fig. 1 b). It clearly shows how the C_9_ metabolites reach a maximum intensity at ~ 0.7 L to then decrease. In contrast, the exhalation profile for the longest aldehydes (C_12_ and C_15_) tends to increase systematically with exhaled volume (similarly to CO_2_ profiles). We hypothesize that, as the exhalation maneuver is repeated, the net influx towards bronchial circulation exceeds that outwards. Thus, the partial pressure cannot re-equilibrate in the short lapse in-between exhalations, leading to a constant non-linear decay across the repeated measurements. For 4-hydroxy-2-dodecanal, we observed a deviation from the decaying pattern (Fig. 3 a and ESM Fig. S5). The underlying reason might be that this particular *m/z* channel is dominated by an isomer of 4-hydroxy-2-dodecanal. It is important to note at this point that this is a limitation of SESI-HRMS, as discrimination of isomers is sacrificed by the possibility of performing real-time analysis.

In order to further connect the theoretical explanation as to why *λ*_*b:a*_ ultimately modulates the decay in signal intensity due to gas exchange in the airways, Fig. 3 b (and ESM Fig. S10) shows the experimental average breath-signal difference between the last and first exhalation, as a function of predicted *λ*_*b:a*_. These *λ*_*b:a*_ values were estimated by fitting the *λ*_*b:a*_ for all aldehydes reported by Kramer et al. [63] (ESM Fig. S9). It reveals a clear trend, whereby for the longest chain (C_16_) the difference tends to increase during the repeated measurements. This is especially evident for 4-hydroxy-2-hexadecenal (ESM Fig. S6). In contrast, as the chain length decreases (and thus *λ*_*b:a*_ increases), the breath-signal difference decreases to finally reach a plateau of Δ −20% to −40% at C_11_. The fact that this clear trend occurs in the transition boundaries between 10 < *λ*_*b:a*_ < 100 suggests that indeed this may be due to the different regions of the respiratory system where these series of compounds exchange: from alveoli for C_16_ to airways for C_8_, with a mixed exchange situation for intermediate species. Further work is required to confirm this hypothesis and whether this could be further exploited to infer physiological information of the respiratory system, for example, complementing other tests such as the multiple-breath washout test to measure abnormal ventilation distribution between well- and poorly ventilated lung regions.

Despite that the first exhalation may reflect more accurately systemic concentrations for metabolites with high blood-air partition coefficients, we recommend to sample at least ten replicate exhalations and compute breath-signals considering only the last three exhalations, thus capturing the steady state. When doing so in the example shown in Fig. 1 b, the median CV (IQR) for the 27 aldehydes was 4.1% (1.5%), which approaches the technical variability of ~ 3% measured with standard β-pinene vapors. However, for pediatric patients and patients suffering from respiratory diseases, this may prove difficult. For this reason, in order to determine an upper bound of expected variability, we have evaluated here the variability of breath metabolites across all subjects considering only six exhalations and excluded the first three maneuvers to the breath-signal for metabolites. When doing so, we found that the median CV (IQR) for the aldehydes studied here was 6.7% (5.5%). Table 1 lists the intra-subject CVs for the 27 aldehydes studied here.

### Flow dependency

**Fig. 4.**
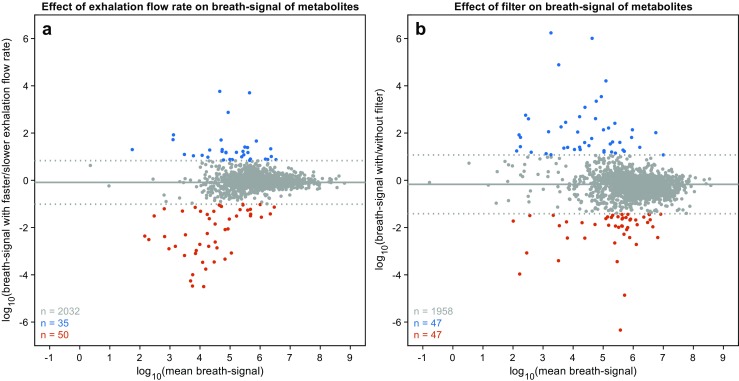
Evaluation of breath mass spectra at varying exhalation flow rates and using spirometry filters. **a** Breath-signals of exhaled metabolites are independent of exhalation flow rate. As seen by the comparison of signals from two experiments with slower (9.8 ± 0.1 L/min) and faster (12.0 ± 0.3 L/min) exhalation flow rates. **b** Use of filters does not significantly affect the breath-signals of exhaled metabolites. As seen by the comparison of signals from two experiments with and without the presence of an antibacterial/antiviral filter. In both panels, solid gray horizontal line represents the mean and dotted gray horizontal lines represent mean ± 1.96 × SD.

Some studies indicate that the exhalation maneuver itself can in some cases alter the metabolic profile, hence providing misleading results [32]. For this reason, we further investigated whether the exhalation flow rate of our protocol had an impact on the breath-signal of the exhaled metabolites. Flow resistance of the device was as low as 3 mbar × min/L, mean ± SD exhalation flow rates of all the experiments performed in this study (*N* = 104) was 10.6 ± 0.9 L/min (ESM Fig. S11) and typical exhaled volumes were in the order of 3 L (i.e., 15–20 s of exhalation). It is important to note that this maneuver is far less invasive and easy to perform than a classical spirometry, whereby the forced expiratory volume in one second (FEV_1_) can typically be 4 L in adults. This implies exhalation flow rates around 25 times higher than the maneuver used in our experiments. It has been shown that such forced expiration maneuvers can lead to substantial changes in exhaled CO_2_ and other metabolites [32]. The fact that no significant changes in the CO_2_ levels were observed suggests that the maneuver does not induce hyperventilation [42]. In order to determine whether there was any dependency with the exhalation flow rate, we explored the impact of exhaling at two flow rates, one at the lower end and another one at the upper end of the distribution of exhalation flow rates measured for all participants (ESM Fig. S11). Figure 4 a shows the comparison of two measurements from the same subject at a lower flow rate (9.8 ± 0.1 L/min) and consecutively at a higher flow rate (12.0 ± 0.3 L/min). Bland-Altman plot for log-transformed variables shows that the breath-signal of metabolites is independent of the exhalation flow rate. The mean of log_10_(ratio) was found to be − 0.09. As expected, only ~ 4% of low-intensity ions lie outside the mean ± 1.96 × SD bands. We therefore conclude that the range of flow rates between 9 and 12 L/min are suitable for breath metabolomics using our particular configuration.

### Antibacterial/antiviral spirometry filter

Patient and operator safety and hygiene are crucial factors to take into account in the clinics. For this reason, the interface between the patient and the breath analysis platform is through a disposable barrier filter, as the ones routinely used for pulmonary function testing. This is a new element incorporated in this device to allow for measuring patients with suspected respiratory infectious diseases. Until now, our system featured a mouthpiece filter used for alcohol breath tests, which would not be suitable to investigate contagious respiratory diseases. In a separate set of experiments, we examined whether these aerosol filters may have an impact on the detected metabolites. To do so, we compared the breath-signal of the same subject exhaling through the filter and subsequently exhaling without the filter. Figure 4 b shows the resulting comparison, represented as a Bland-Altman plot for log-transformed variables. There appears to be a small bias towards lower intensities by the use of the filter, as the mean of log_10_(ratio) was found to be − 0.17. Moreover, only 4.6% of the signals fell outside the mean ± 1.96 × SD boundaries. Globally, these results are consistent with previous studies suggesting that SESI-MS breath spectra using and removing aerosol filters look alike [64]. We therefore conclude that, while the antibacterial/antiviral filters incorporated in our system may partially suppress some signal intensities, they represent a good compromise to protect the system and the operator from pathogens and to preserve the quality of the mass spectral readout of exhaled metabolites.

### Instrumental time drift

**Fig. 5:**
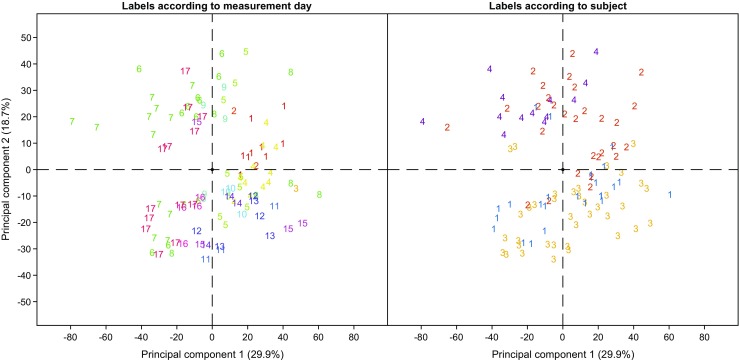
Variability of SESI-HRMS breath mass spectra are dominated by inter-individual differences, rather than by batch effect**.** PCA score plot of all measurements with labels according to measuring day (left) and subject number (right). Grouping according to subject number is more evident than by measurement date.

Instrumental time drifts and batch effects are a common problem in untargeted metabolomics [65, 66]. This can be especially critical in clinical studies as patient recruitment typically runs over several months/years. In order to assess whether our system showed any significant batch effect due to the date of measurement, we visualized our data using principal components analysis (PCA). Figure 5 shows the resulting plot for the first two components, whereby the labels on the left-hand side correspond to a total of 17 measuring days spanning across 1 month. No clustering according to measuring day is evident, suggesting that the variance explained by these two components (48.6% in total) cannot be attributed to a batch effect. Note that no special cleaning procedures, apart from flushing the ion source with hot nitrogen overnight, were performed during this month of operation. In contrast, on the right-hand side of Fig. 5, the same score plot is shown whereby the labels now indicate the subject number. Grouping based on the subject number is much more evident. For example, subjects 1 and 3 cluster together suggesting a significantly different exhaled metabolic phenotype than subjects 2 and 4. This is also consistent with previous studies suggesting the existence of stable individual-specific metabolic traits [67–69]. The same picture emerged when we considered the 27 representative aldehydes (ESM Fig. S12). In order to provide a more objective assessment of whether significant differences exist across subjects for these metabolites, we conducted an ANOVA test followed by post hoc multiple comparison using a Bonferroni method (Table 2).

This univariate approach revealed significant differences in the breath-signal of exhaled aldehydes. Overall, the median (IQR) relative difference between individuals (considering only those *p* ≤ 0.05) was 48.2% (39.3%). This is consistent with inter-subject variability in blood concentrations for these particular compounds. For example, Mak et al. [70] reported CVs for 4-hydroxy-nonenal from eight healthy individuals of 95.8%. In our case, mean differences between subject 1 and 2 were of 42.4% for this particular compound. It is therefore evident that the inter-subject biological variability is greater than intra-subject variability, and is consistent with the variability expected in blood levels.

### Fatty aldehydes as surrogate markers of oxidative stress

**Fig. 6.**
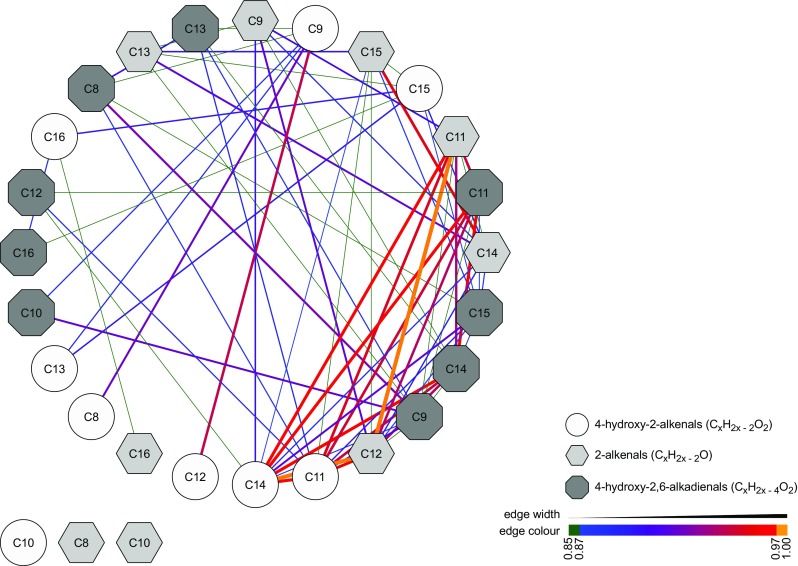
Positive correlation among aldehydes suggests a common origin of mechanism of generation. Correlation network (considering Spearman’s *r* ≥ 0.85) with an average node degree of 4 ± 2. Note that 4-hydroxy-2-decenal, 2-octenal and 2-decenal do not pass the correlation cutoff and hence are shown at the bottom-left side. Node shape and color are based on the classes of aldehydes, whereas edge width and color depends on the correlation coefficient, as shown in the legend at the bottom-right side.

Fatty aldehydes were chosen as metabolite models for this study as they are related to lipid peroxidation and oxidative stress. Oxidative stress is the trigger for the production of fatty aldehydes, such as 4-hydroxy-2-nonenal, in human metabolism [71]. Abnormally elevated values (factor two to three as compared to controls) of some of the aldehydes studied here have been associated with pathologies such as congestive heart failure [70]. Strong associations between series of metabolites, i.e., in terms of correlations, might be an indication for a common metabolic pathway, as already shown previously for series of omega-oxidation end-products of aliphatic fatty acids [52, 72] and aminoacids [73]. In an attempt to visualize whether an interplay between the different series of fatty aldehydes may be captured by breath analysis, we computed correlation coefficients across all measurements. A first indication suggesting that these metabolites are indeed metabolically connected is given by the fact that all of them showed positive correlations (ESM Figs. S13-15). Thus, all measured subjects had consistent (high or low) breath-signals for all 27 metabolites. One could argue that this might be an artifact as a result of different performance of the system during the different days (i.e., consistently high- or low-intensity mass spectra). However, this can be ruled out as we found that these aldehydes consistently correlated with each other, but not with the rest of the over 2,000 features considered in the breath mass spectra (ESM Fig. S16). Only around 2% of the pair-wise correlations for all features correlated with *r* ≥ 0.85 with the aldehydes. We therefore conclude that the observed associations for these families of compounds should encode a biological meaning. Figure 6 shows the resulting correlation network for the aldehydes. Most of the aldehydes are indeed linked with a mean ± SD degree of 4 ± 2 (*r* ≥ 0.85). This is to be expected from the metabolic point of view, as aliphatic aldehydes in humans are largely produced by a cascade of catabolic metabolism of several lipids [71]. In particular, peroxidative cleavage of polyunsaturated fatty acids by reactive oxygen species is the mechanism behind a complete series of aldehydes as those studied, including short- and medium-chain aldehydes, or hydroxy-alkenals.

## Conclusions

Summing up, we presented here a series of instrumental developments aiming to standardize sampling and analysis of expired metabolites by real-time SESI-HRMS. This analytical platform was tested using a constant infusion of β-pinene vapors in the ppb range resulting in a technical variability within 3%. We then tested the system during a series of repeated breath measurements from four healthy individuals. Real-time display of CO_2_, exhalation flow rate, and exhaled volume to the subjects during the exhalation maneuver enabled a variability for these variables within 5%. We found no evidence that the exhalation maneuvers would induce hyperventilation, nor that the exhalation flow rates and mouthpiece filter used would have any significant impact on the quality of the metabolic breath print. We also did not find any evidence of obvious batch effect. However, despite these indications of exhalation maneuver control and reproducibility, we observed a systematic decay in the signal intensity of the shorter aldehydes across all measurements for all subjects. This compound-specific and individual-independent pattern has been rationalized as a result of the different locations of the respiratory system where the aldehydes may exchange. We hypothesize that shorter aldehydes exchange within the airways (with the bronchial blood), and longer ones primarily in the alveoli (with the pulmonary blood). Although the first exhalation may correlate better with systemic aldehyde concentrations, we recommend the collection of at least six replicate exhalations per subject and exclude the first three from the analysis. Caution should be taken when interpreting results from such measurements, especially for shorter species. Taking into account these measures, we found intra-subject variabilities is in general much lower than inter-subject variability for the aldehydes studied (6.7% vs 48.2%). Such inter-subject differences are consistent with reported variability of such aldehydes in blood. Moreover, we found that all 27 aldehydes strongly positively correlated with each other, which is to be expected due to their common metabolic origin in humans. Overall, we conclude that this breath analysis platform and procedures described herein meet the required standards to conduct breath metabolomics studies in multi-center clinical studies. Further work to interrogate exhaled breath using this analytical platform in two different clinical settings is ongoing.

## Electronic supplementary material

ESM 1 (PDF 4140 kb)

## Data Availability

The raw mass spectra and Exhalion files of the real-time breath measurements are available from the MetaboLights (https://www.ebi.ac.uk/metabolights) repository (accession number MTBLS842).
