# Supplementary material for: Standardization procedures for real-time breath analysis by secondary electrospray ionization high-resolution mass spectrometry
Source: Anal Bioanal Chem. 2019 Apr 15;411(19):4883–98. doi: 10.1007/s00216-019-01764-8 (PMC6611759; doi:10.1007/s00216-019-01764-8)
Supplement: Supplementary file 1 — (PDF 4140 kb) [file 216_2019_1764_MOESM1_ESM.pdf]

Analytical and Bioanalytical Chemistry

Electronic Supplementary Material

Standardization procedures for real-time breath analysis by secondary electrospray ionization high-resolution mass spectrometry

Kapil Dev Singh, Georgi Tancev, Fabienne Decrue, Jakob Usemann, Rhea Appenzeller, Pedro Barreiro, Gabriel Jaumà, Miriam Macia Santiago, Guillermo Vidal de Miguel, Urs Frey, Pablo Sinues

Sample Size Calculation

The width of the 95% confidence interval (CI) for the population within-subject standard deviation (SD) is defined as:

$$Width\ of\ 95\%\ CI = \frac{1.96 * SD}{\sqrt{2m(n-1)}} \tag{1}$$

Where, m is the number of subjects and n is the number of repeated measurements per subject. Thus, one can estimate it to within some fraction of the population value.

$$Width\ of\ 95\%\ CI = \frac{1.96 * SD}{\sqrt{2m(n-1)}} = p * SD \tag{2}$$

This is an equation with two unknown quantities: *m* and *n*. There are multiple combinations of *m* and *n* which will give the required precision (*p*). **Table S1** shows the solution of equation 2 for six different scenarios considering three, four, and five subjects and target precision of 5% and 10%. In this study we aimed to enroll 3 to 5 subjects to achieve a precision of at least 10% (*i.e.* 65 to 39 measurements per subject).

Table S1 Sample size calculation table based on solution for equation 2. At the end we enrolled 4 subjects with at least 49 exhalations to achieve at least 10% precision

Number of	Number of replicates (n) per subject	
Subjects (m)	5% precision	10% precision
3	257	65
**4**	193	**49**
5	155	39

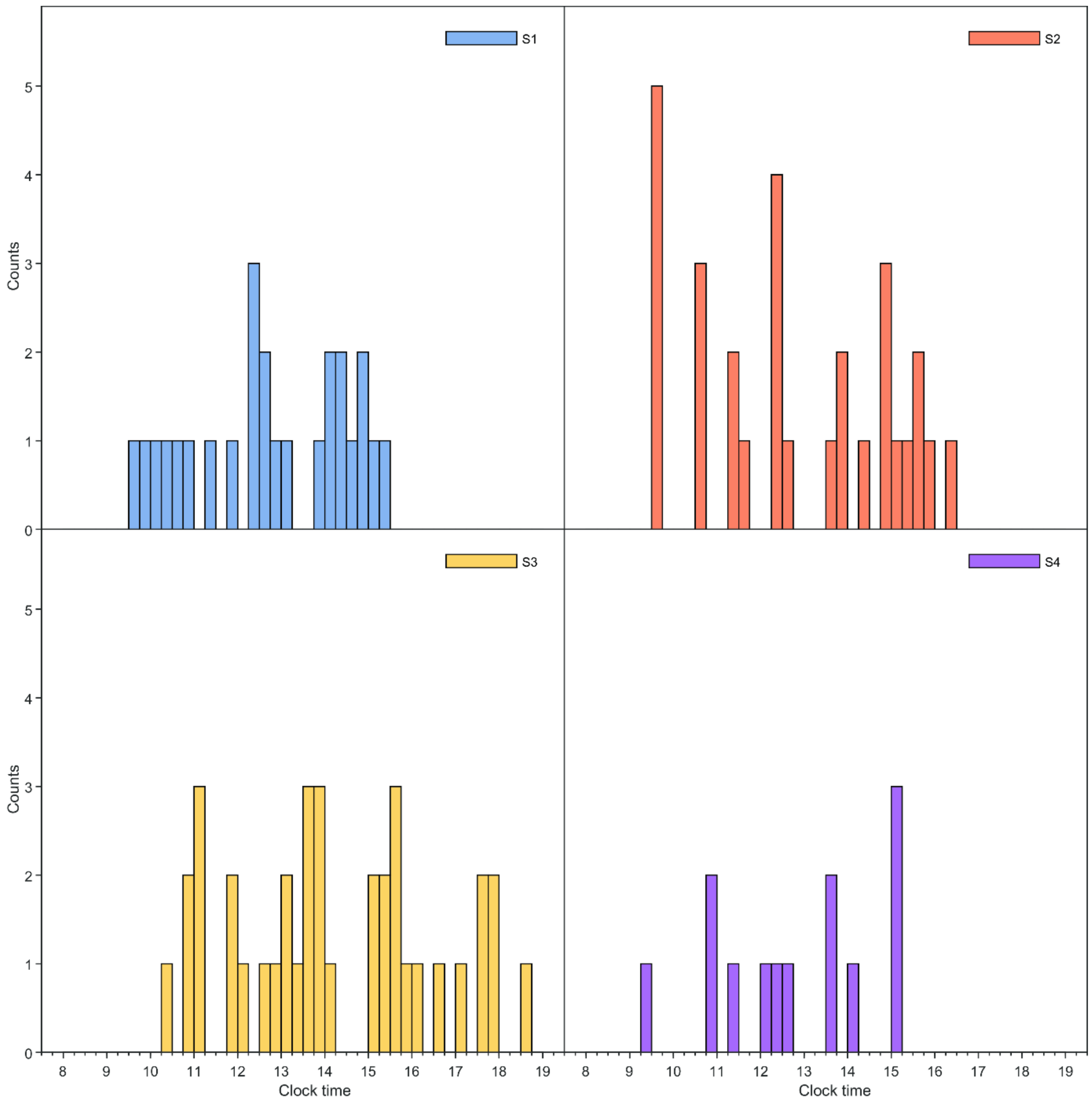

Fig. S1 Inter-individual difference observed are unlikely to be explained by circadian rhythms, as the sampling time was similar for all participants. The histograms depict the sum of all measurements as a function of measurement time for each subject

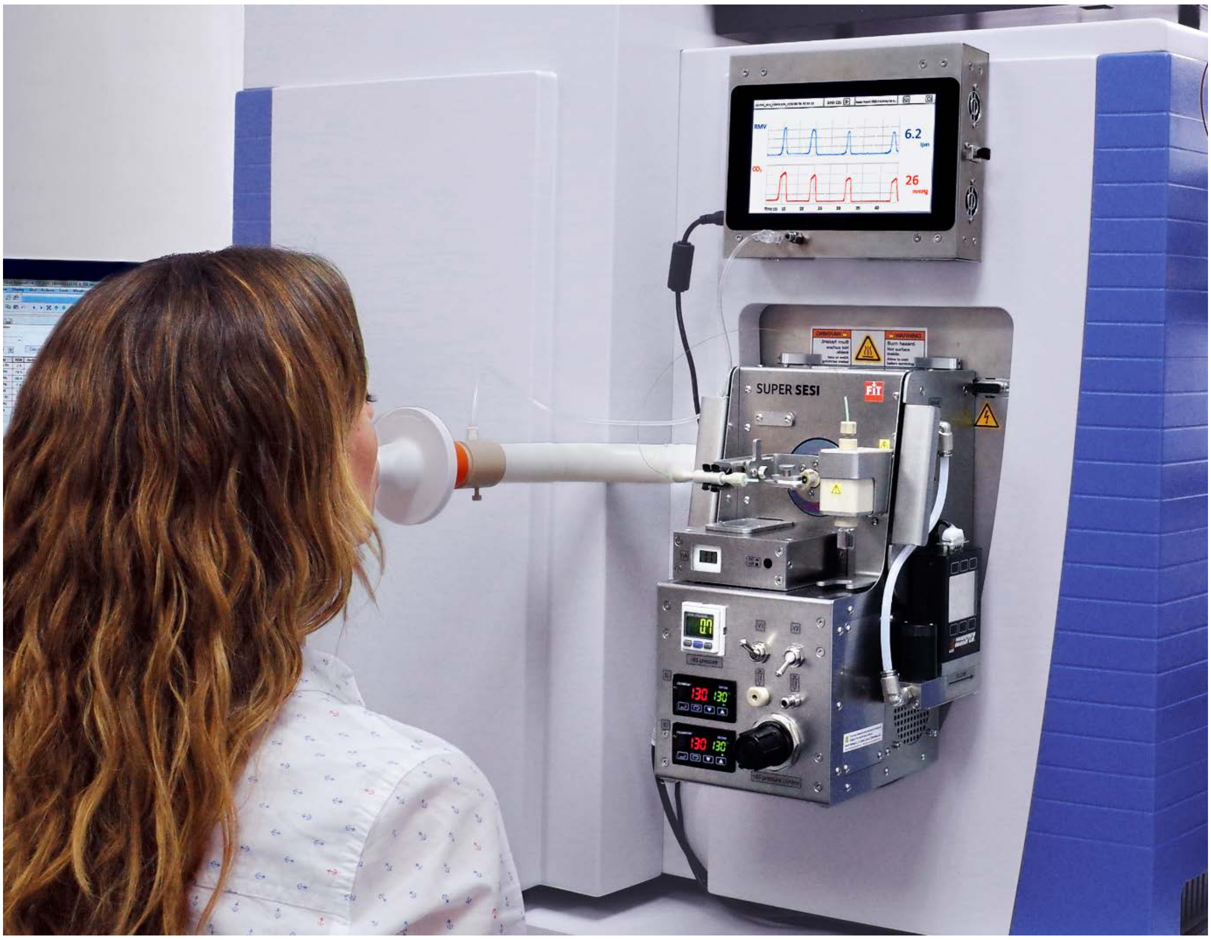

Fig. S2 A pictorial representation of a subject exhaling into the real-time breath analysis platform used in this study

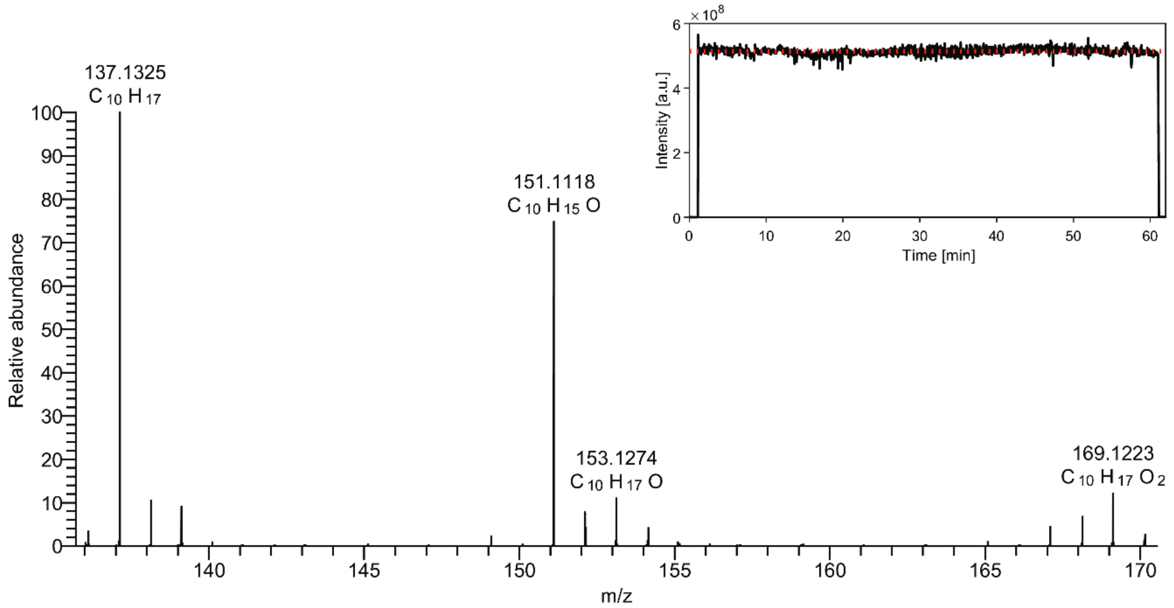

Fig. S3 Technical CV as measured by using β-pinene standards is within 3%. The mass spectrum shows the expected protonated β-pinene at *m/z* 137.1326 ($C_{10}H_{17}$), along with some oxidized species ($C_{10}H_{15}O$, $C_{10}H_{17}O$, and $C_{10}H_{17}O_2$). Note that high resolution and accuracy of Q Executive Plus enables unambiguous molecular formulae assignment. Inset shows the summed ion current for β-pinene and the oxidized species

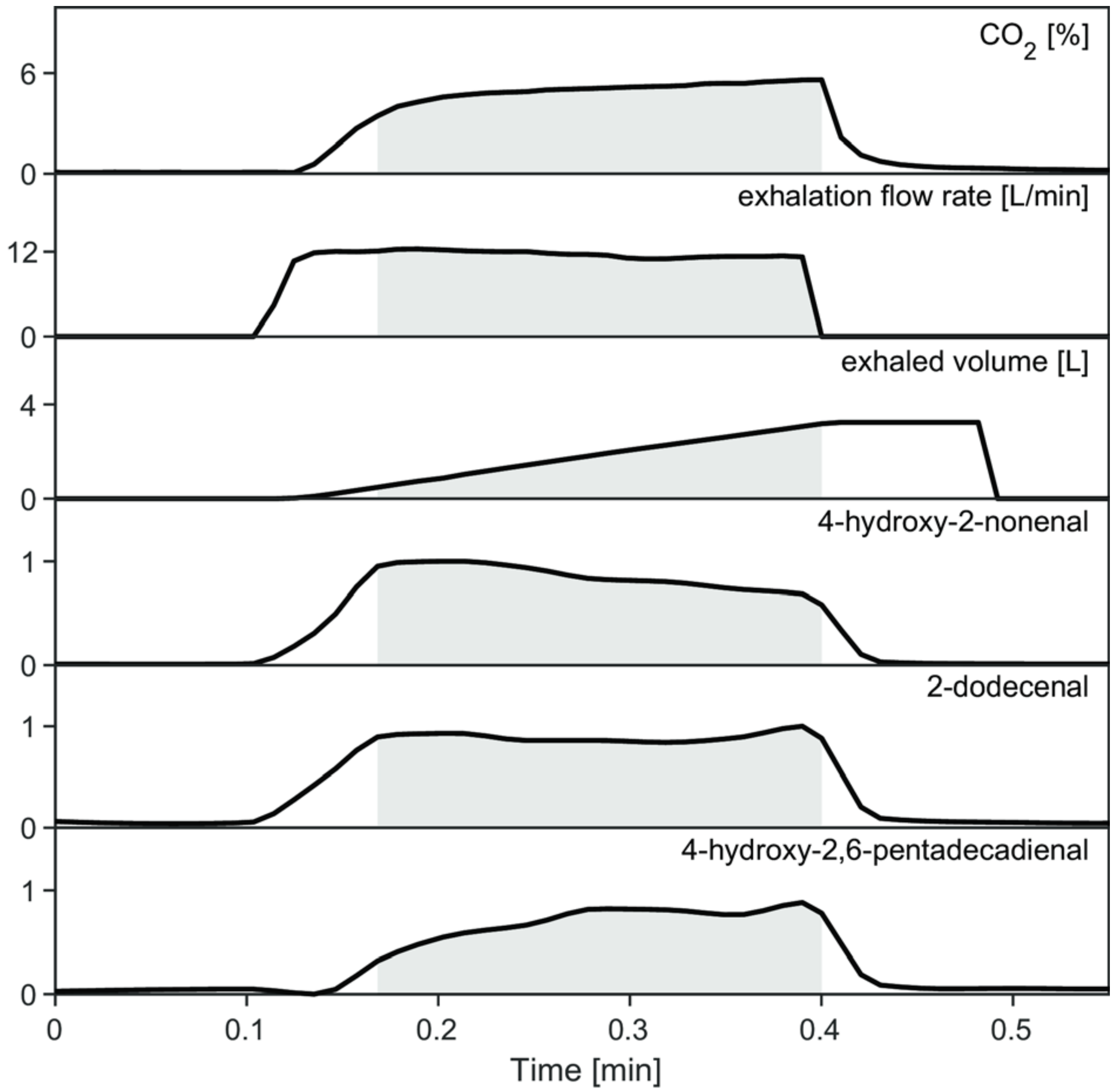

Fig. S4 Zoomed-in view of first exhalation from Fig. 1B. The grey areas here and also in **Fig. 1B**, show the time windows whereby CO_2 levels were above 3%. Note that the exhaled volume (computed by real-time integration of the exhalation flow rate) holds its value for few seconds after the end of the exhalation. This does not have any effect on analysis because we calculated the exhaled volume based on the bounds within 3% CO_2

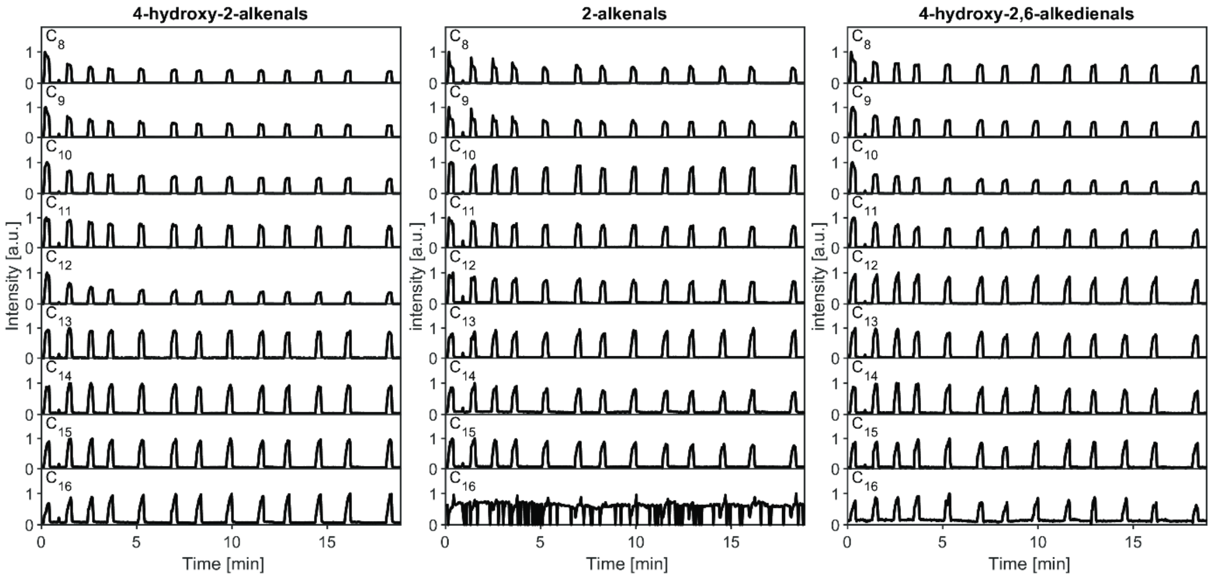

Fig. S5 Selected aldehydes show a chain length-dependent and class-independent decay of signal intensity over time within an experiment. Note the deviation of 4-hydroxy-2-dodecenal from the regular pattern, which might be due to an interfering peak. 2-hexadecenal (C_{16}) was not detected in this particular experiment

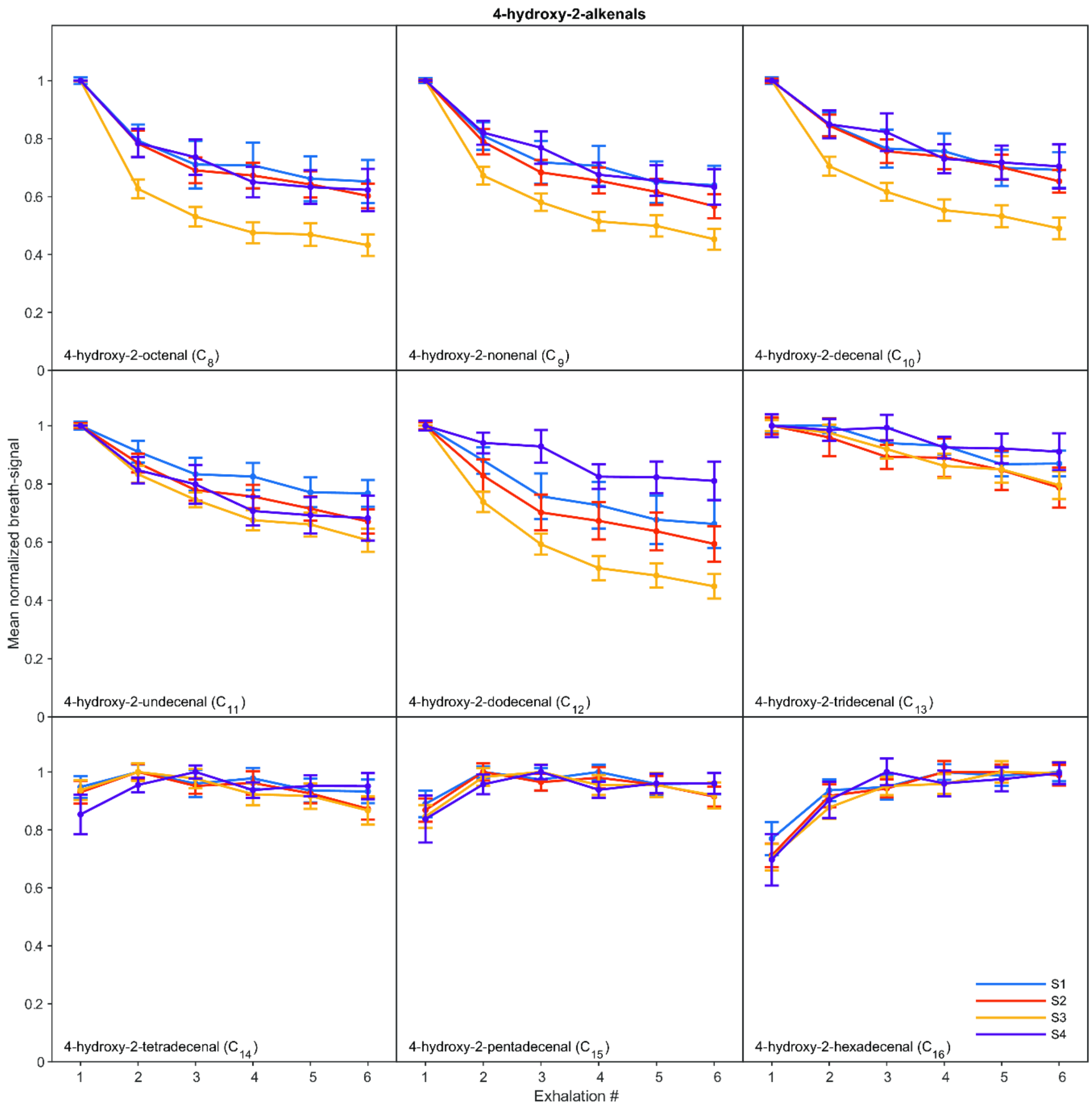

Fig. S6 Mean normalized breath-signal with error bars representing 95% CIs for 4-hydroxy-2-alkenals from four subjects in 104 experiments. Note the deviation of 4-hydroxy-2-dodecenal from the regular pattern, which might be due to an interfering peak

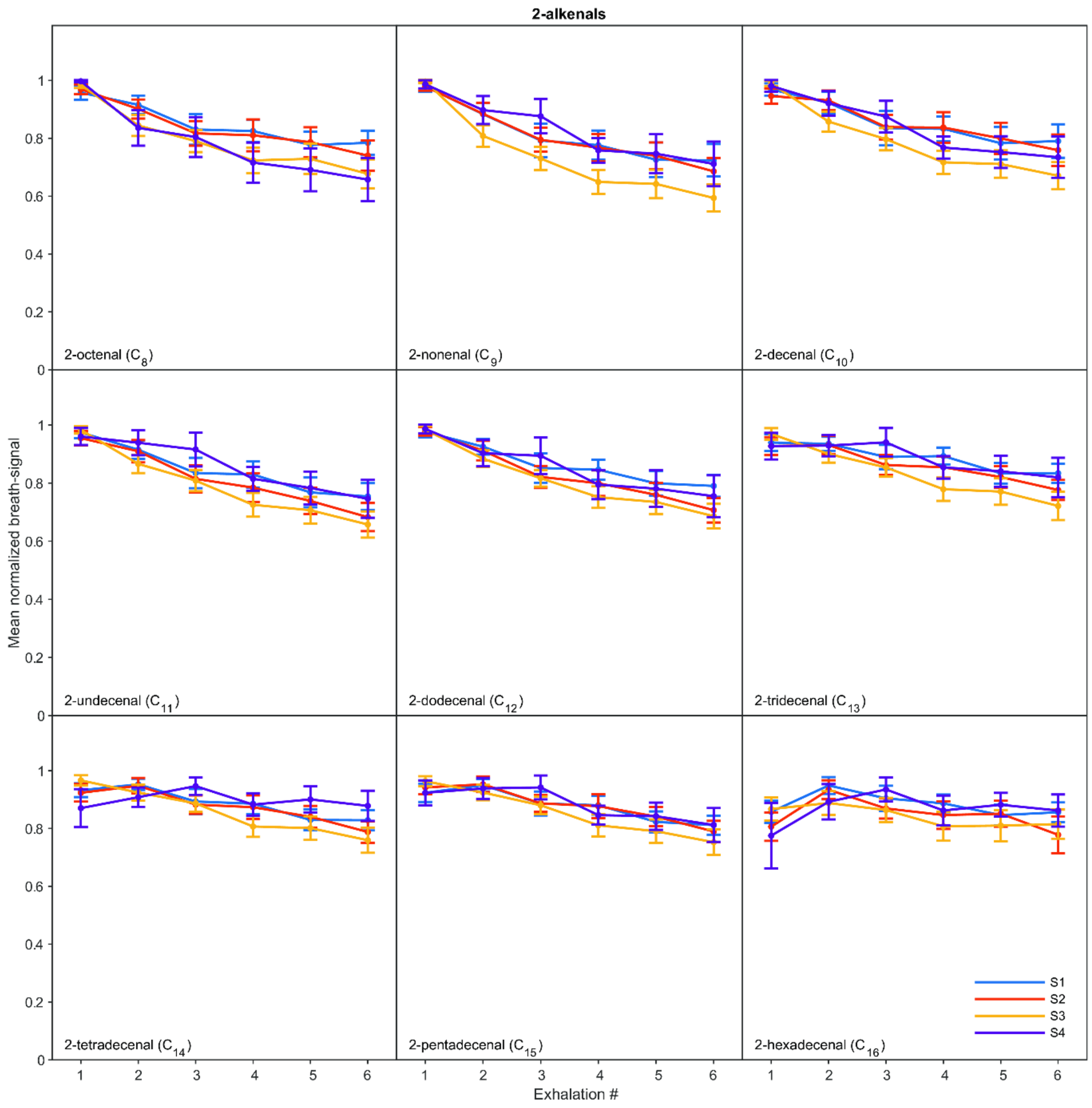

Fig. S7 Mean normalized breath-signal with error bars representing 95% CIs for 2-alkenals from four subjects in 104 experiments

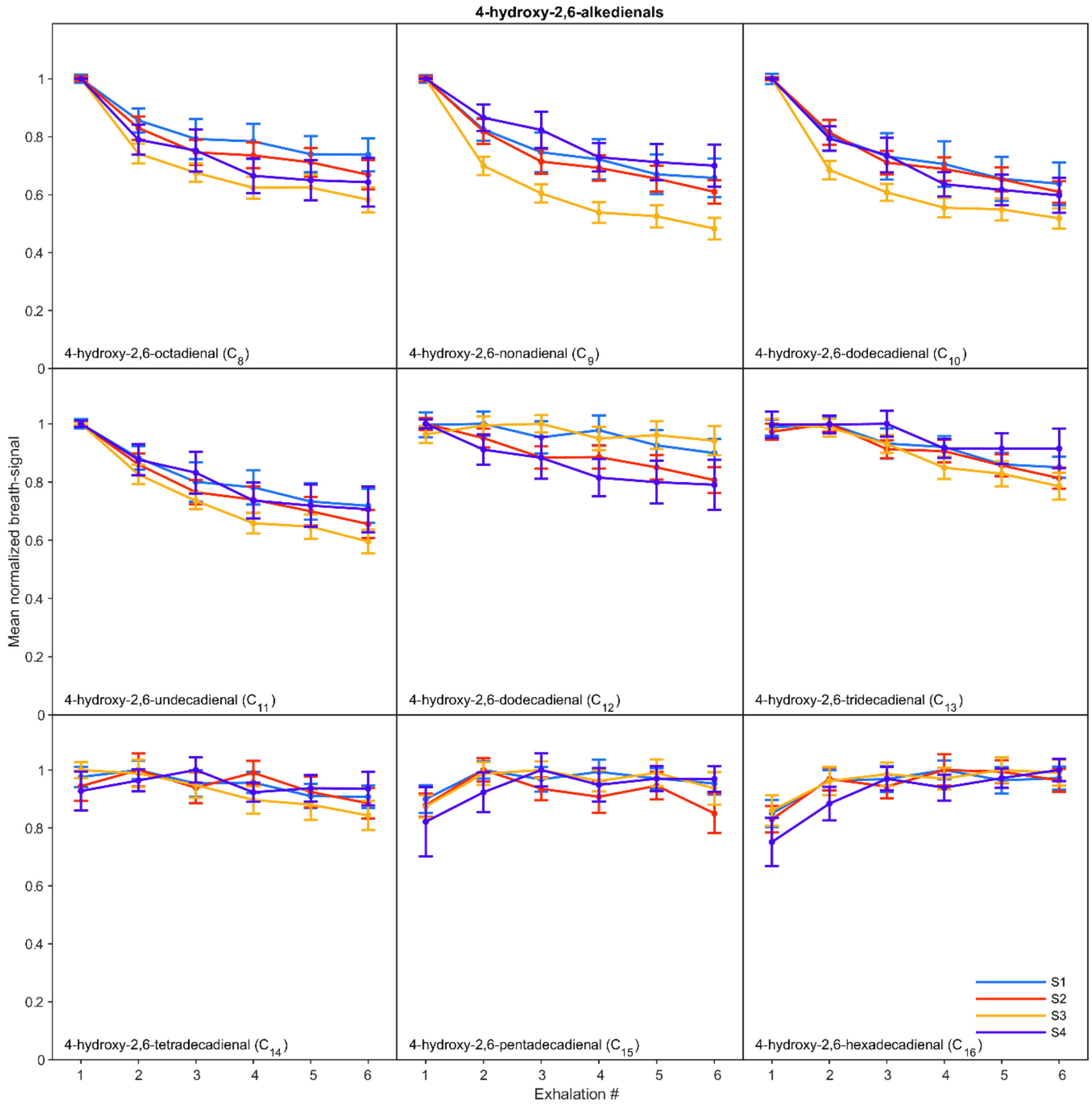

Fig. S8 Mean normalized breath-signal with error bars representing 95% CIs for 4-hydroxy-2,6-alkedienals from four subjects in 104 experiments

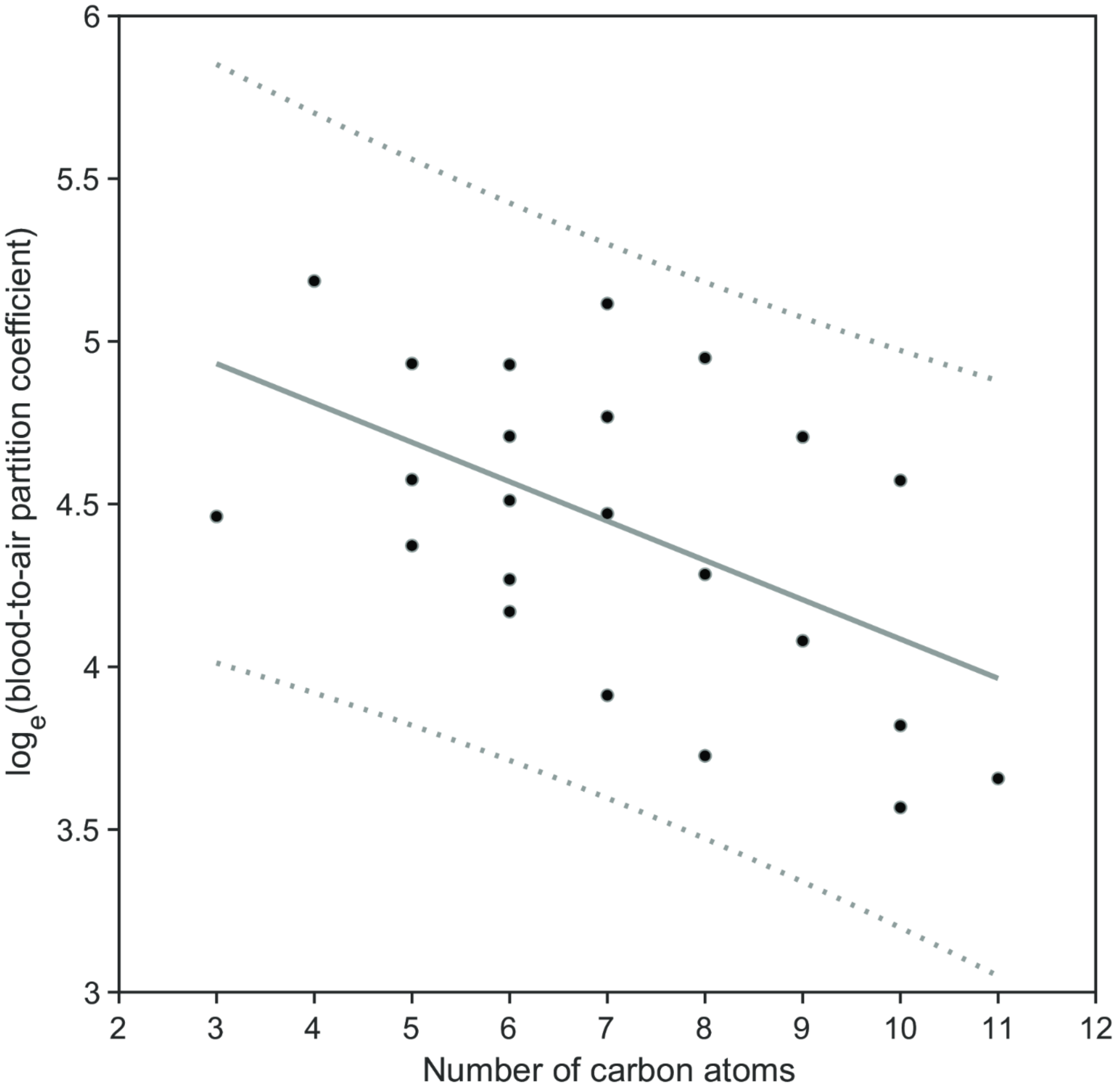

Fig. S9 Dependency of blood-to-air partition coefficient on chain length for a series of aldehydes. Data extracted from Kramer *et al.* [1]

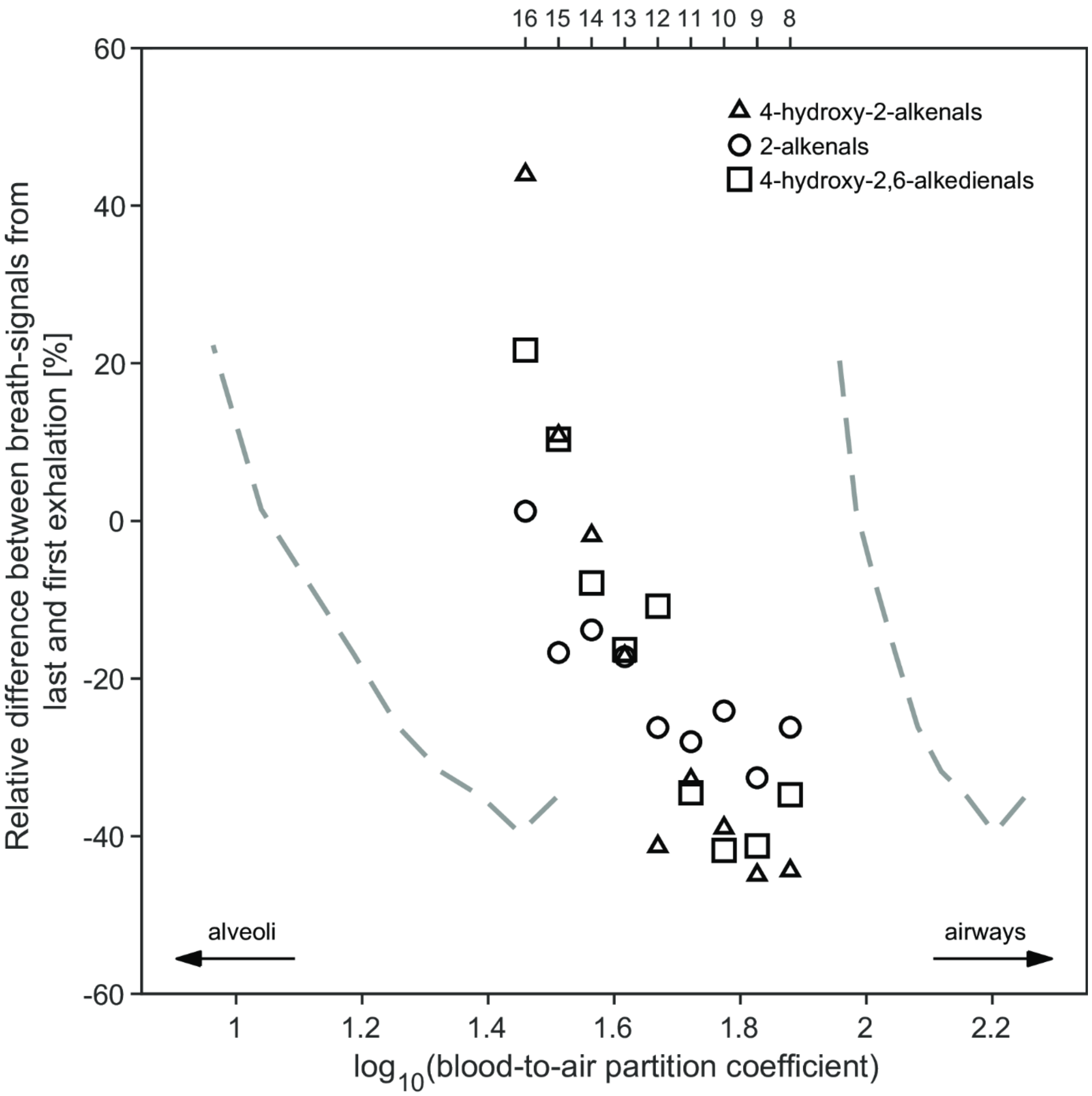

Fig. S10 Exhalation profile of breath metabolites indirectly depends on their blood-to-air partition coefficient. Same as Fig. 3, but x-axis is on $\log_{10}$ scale to show the complete range of 95% CIs

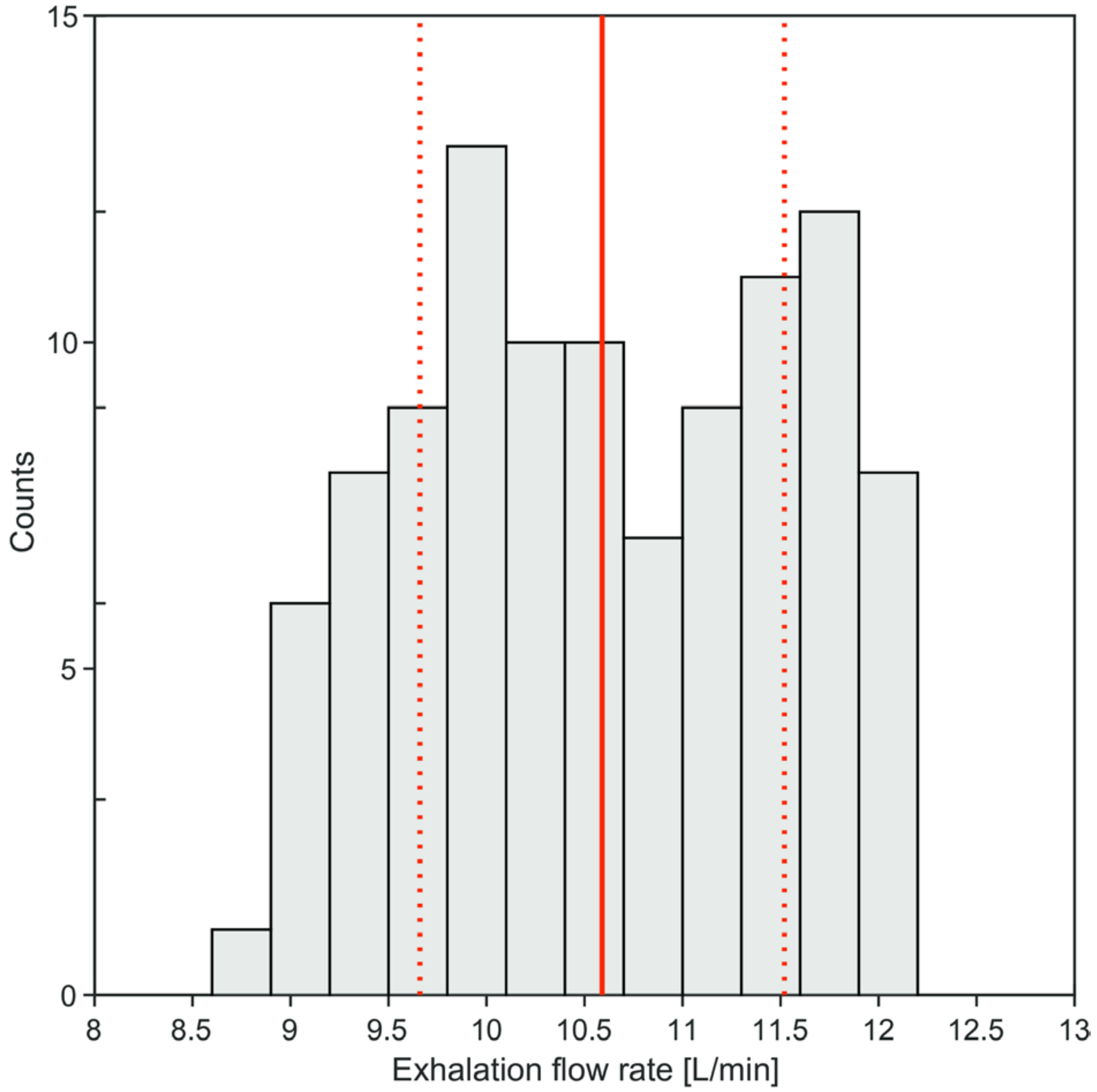

Fig. S11 Distribution of the exhalation flow rates (L/min) from all the experiments of this study. Solid red vertical line represents the mean and dotted red vertical lines represent SD

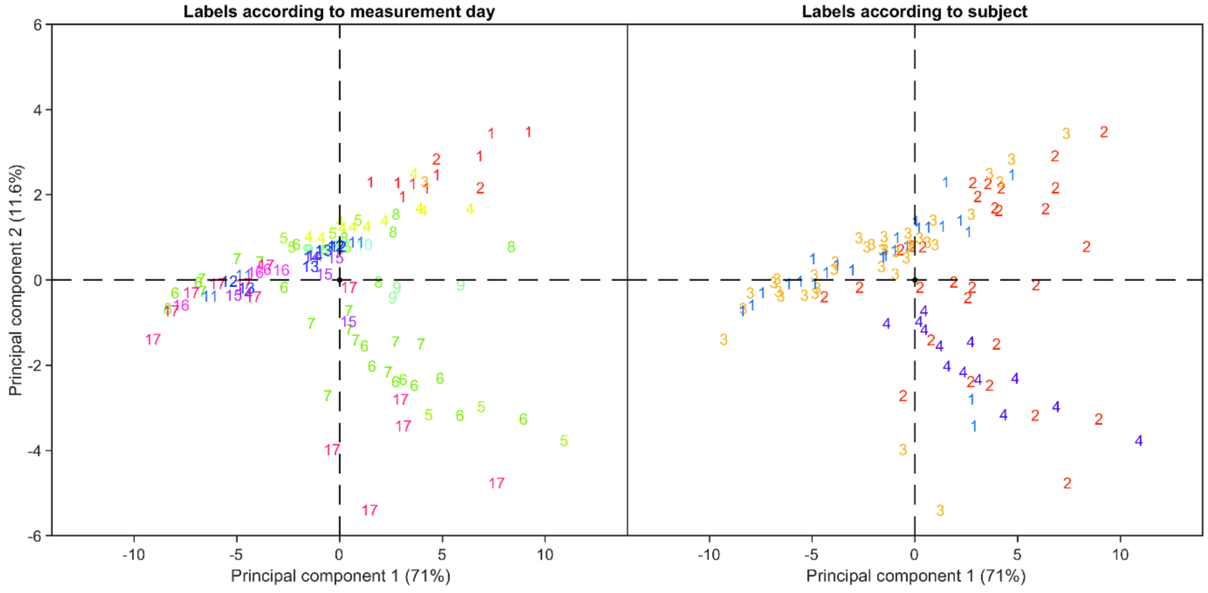

Fig. S12 Variance observed for the 27 aldehydes studied in this work is better explained by the subject number rather than measurement day (see also Fig. 5 for more details)

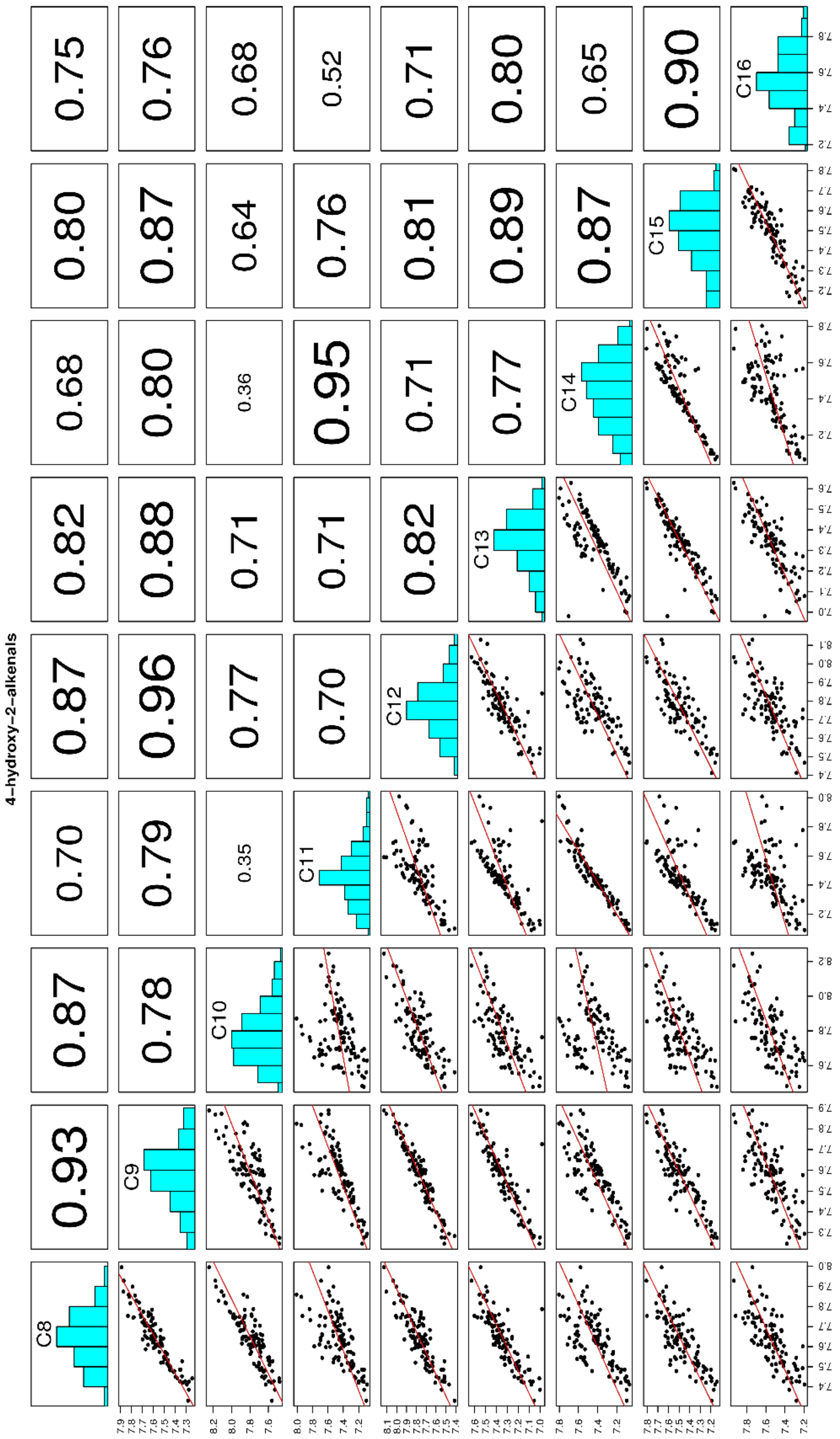

Fig. S13 Correlation matrix for the breath-signals (log_{10} scaled) of 4-hydroxy-2-alkenals class aldehydes from 104 experiments. The solely positive correlation among all molecules indicates a common mechanism by which they are generated. Diagonal shows distribution of breath-signals from 104 experiments for C_8-C_{16} aldehydes members of class 4-hydroxy-2-alkenals. Scatter plots between members are shown below the diagonal and Pearson correlation coefficients between members are shown above the diagonal

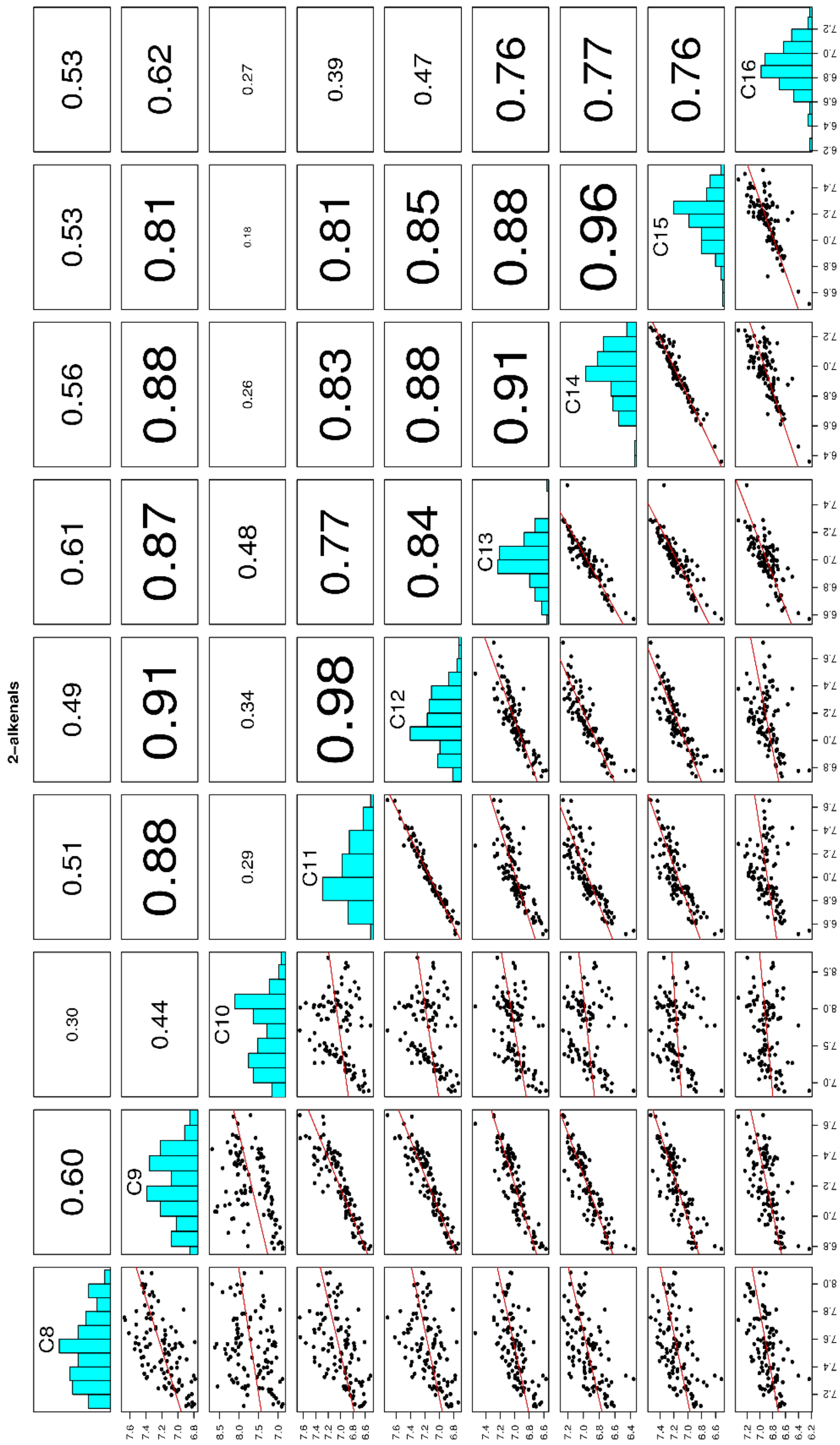

Fig. S14 Correlation matrix for the breath-signals (log_{10} scaled) of 2-alkenals class aldehydes from 104 experiments. The solely positive correlation among all molecules indicates a common mechanism by which they are generated. Diagonal shows distribution of breath-signals from 104 experiments for C_8-C_{16} aldehydes members of class 2-alkenals. Scatter plots between members are shown below the diagonal and Pearson correlation coefficients between members are shown above the diagonal

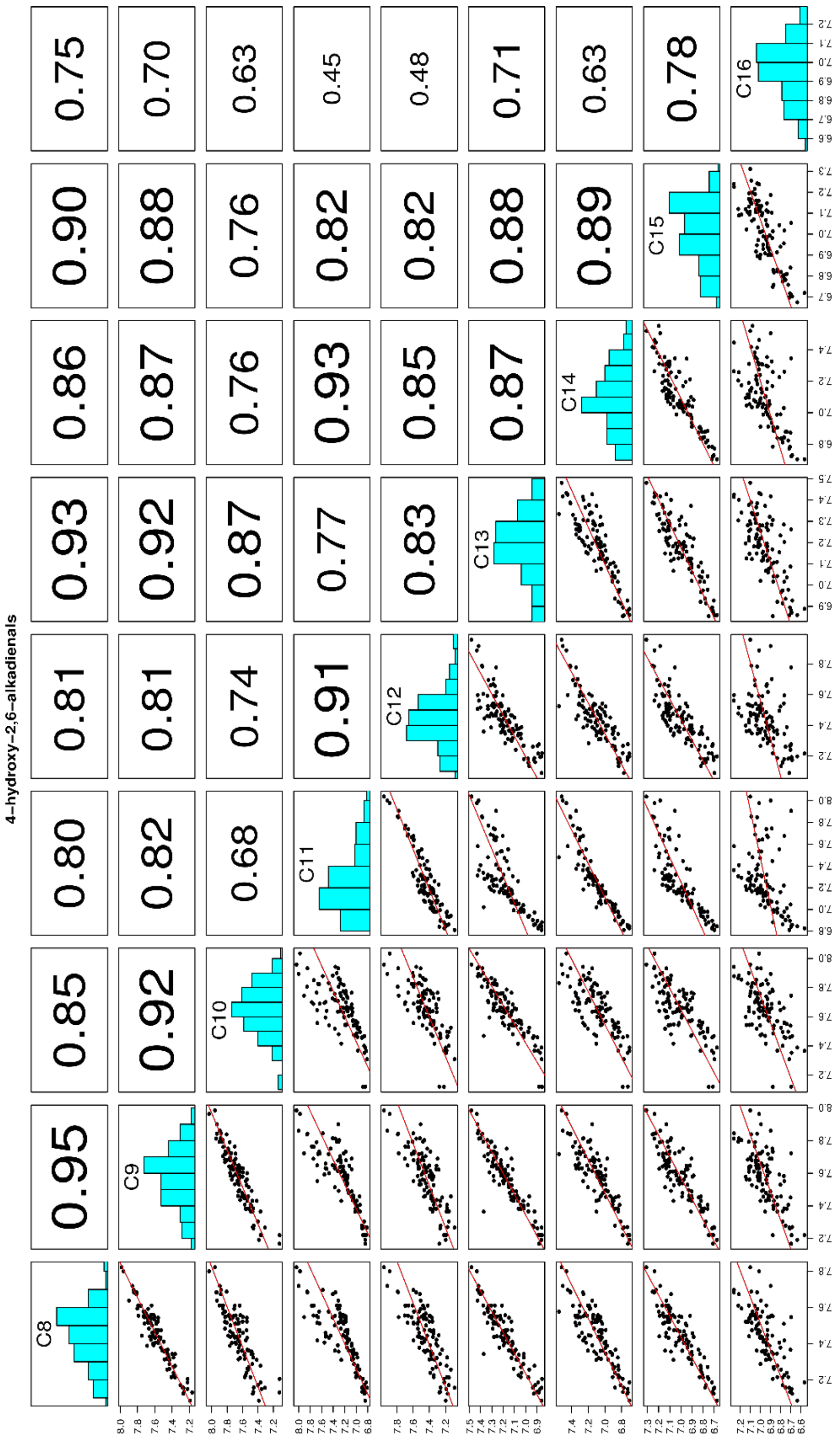

Fig. S15 Correlation matrix for the breath-signals (log_{10} scaled) of 4-hydroxy-2,6-alkadienals class aldehydes from 104 experiments. The solely positive correlation among all molecules indicates a common mechanism by which they are generated. Diagonal shows distribution of breath-signals from 104 experiments for C_8-C_{16} aldehydes members of class 4-hydroxy-2,6-alkadienals. Scatter plots between members are shown below the diagonal and Pearson correlation coefficients between members are shown above the diagonal

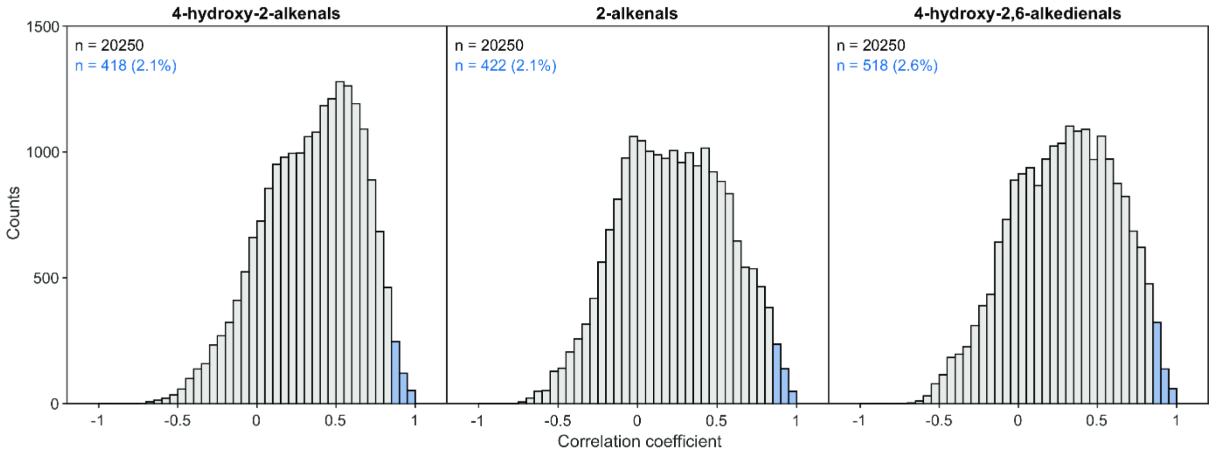

Fig. S16 Selected aldehydes from three classes correlates strongly with each other but not with the rest of the detected features. Data shown are the Spearman correlation coefficient of aldehydes from each class with all quantified features (leading to total 20,250 pairwise correlation values). Bars highlighted in blue shows the fraction of correlation higher than 0.85

References

1. Kramer C, Mochalski P, Unterkofler K, Agapiou A, Ruzsanyi V, Liedl KR. Prediction of blood:air and fat:air partition coefficients of volatile organic compounds for the interpretation of data in breath gas analysis. J Breath Res. 2016;10(1):Artn 017103. https://doi.org/10.1088/1752-7155/10/1/017103.
